# Proteome Response of Chicken Embryo Fibroblast Cells to Recombinant H5N1 Avian Influenza Viruses with Different Neuraminidase Stalk Lengths

**DOI:** 10.1038/srep40698

**Published:** 2017-01-12

**Authors:** Yongtao Li, Fan Ming, Huimin Huang, Kelei Guo, Huanchun Chen, Meilin Jin, Hongbo Zhou

**Affiliations:** 1 State Key Laboratory of Agricultural Microbiology, College of Veterinary Medicine, Huazhong Agricultural University, Wuhan, 430070, P.R. China; 2 College of Animal Husbandry & Veterinary Science, Henan Agricultural University, Zhengzhou, 450002, P.R. China

## Abstract

The variation on neuraminidase (NA) stalk region of highly pathogenic avian influenza H5N1 virus results in virulence change in animals. In our previous studies, the special NA stalk-motif of H5N1 viruses has been demonstrated to play a significant role in the high virulence and pathogenicity in chickens. However, the molecular mechanisms underlying the pathogenicity of viruses with different NA stalk remain poorly understood. This study presents a comprehensive characterization of the proteome response of chicken cells to recombinant H5N1 virus with stalk-short NA (rNA-wt) and the stalkless NA mutant virus (rSD20). 208 proteins with differential abundance profiles were identified differentially expressed (DE), and these proteins were mainly related to stress response, transcription regulation, transport, metabolic process, cellular component and cytoskeleton. Through Ingenuity Pathways Analysis (IPA), the significant biological functions of DE proteins represented included Post-Translational Modification, Protein Folding, DNA Replication, Recombination and Repair. It was interesting to find that most DE proteins were involved in the TGF-β mediated functional network. Moreover, the specific DE proteins may play important roles in the innate immune responses and H5N1 virus replication. Our data provide important information regarding the comparable host response to H5N1 influenza virus infection with different NA stalk lengths.

The neuraminidase (NA) of influenza A virus, a type II membrane glycoprotein, is one of two major glycoproteins on the virus surface. The NA plays a central role in the release of the virus from infected cells by removing terminal sialic acids from oligosaccharide side chains to which the viral haemagglutinin (HA) binds. The NA protein is a tetramer with a boxlike head comprised of four roughly spherical subunits, as well as centrally attached stalk with a hydrophobic region by which the stalk is embedded in the viral membrane. The NA stalk region varies considerably among different viruses, even within the same subtypes. In 1993, researchers investigated the biologic importance of the NA stalk by artificially generating WSN viruses with a deletion, insertion or mutation of the NA stalk region^1^. The results showed that the length of the NA stalk could be variable and correlated with replication and pathogenesis of influenza virus. Since 1997, the amino acid deletions in the NA stalk also had been found in H5N1 influenza virus under field conditions^2^. Especially, a deletion of five amino acids in the viral NA stalk has been observed in the novel reassortant H7N9 viruses in China^3^. To date, numerous studies on influenza viruses with different NA stalk-motif have been explored and indicated the significant role of NA stalk lengths in virus growth, virulence, and even cross-species transmission^4^,^5^,^6^,^7^,^8^,^9^. However, in terms of host factor, little is known about the molecular mechanism of different pathogenesis of H5N1 viruses with different stalk lengths.

Influenza virus virulence factors, such as HA and NA, typically function through interactions with host proteins, and subsequently contribute to the life cycle of virus. The outcome of influenza virus infection is determined by complex interactions between viral and host factors. Although considerable research efforts have been made to understand the role of NA stalk length in pathogenesis of avian influenza virus, most of these studies have focused on the viruses themselves. Some proteomic analyses had been performed against influenza virus-host models, however, these studies on host response to influenza virus infection primarily targeted human influenza viruses, specifically, only one infection period. For instance, quantitative proteomics has been applied to influenza-host interactions in cells^10^,^11^,^12^,^13^ and several proteomic studies have also been carried out in influenza virus infected animals^14^,^15^.

The previous proteome study on chicken response to H5N1 infection was limited, mainly due to the lack of a chicken protein sequence database and that the analysis was confined to only a single time point for a single viral evaluation^15^. Therefore, a comprehensive understanding of the host response to different viruses with pathogenic diversity is needed. To further look into the pathogenesis mechanism of H5N1 viruses, more attention should be paid to comparable host response to different virus infection, particularly to pair viruses with highly homologous sequences and greatly different virulence. In our previous studies, *in vivo* and *in vitro* experiments of a series of H5N1 viruses with different stalk-motifs showed that the parent virus with short NA stalk-motif (rNA-wt with 20 amino acids deletion from 49th to 68th in the NA stalk) displayed the highest virulence and pathogenesis, while other recombinant viruses with different stalks, especially NA stalkless mutant virus (rSD20) displayed an obviously attenuated phenotype^2^. In this study, we have used 2-dimensional (2-D) electrophoresis based quantitative proteomics to characterize systematically the DE proteins or signaling pathways activated by H5N1 influenza A viruses with different NA stalk lengths in chicken embryo fibroblast (CEF) cells. Herein, we provide evidence that TGF-β signaling cascades are inhibited in rNA-wt infected cells and can be stimulated by rSD20 in cells. We also show that type I interferon (IFN) can be induced by H5N1 influenza virus and can be stimulated more highly in rSD20-infected CEF cells, which might be contributed to some specific DE proteins identified between the two virus-inoculated groups. Our data provide important information regarding the comparable host response to H5N1 influenza virus infection with different NA stalk lengths.

## Results

### Characteristics of two H5N1 influenza viruses infection kinetics in CEF cells

**Figure 1:**
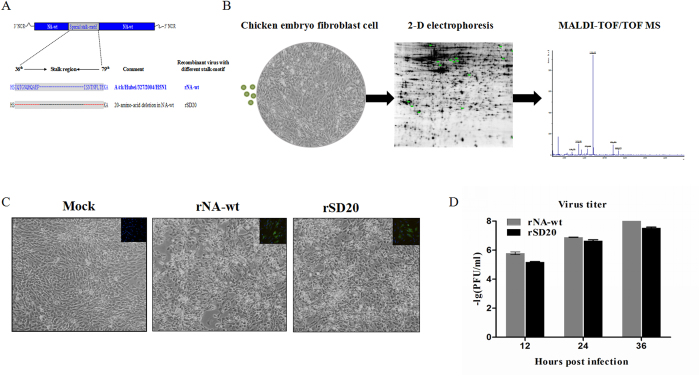
Flow diagram and characteristics of the two H5N1 influenza viruses. (**A**) the parent virus A/chicken/Hubei/327/2004/H5N1 with 20 amino acids deletion from 49^th^ to 68^th^ in NA stalk-motif (designated as rNA-wt) and mutant NA stalkless virus (designed rSD20) were chose for the proteomic study. (**B**) Workflow of the experiment. (**C**) The CPE of infected CEF cells at 24 hpi was showed and the percentage of cells infected by virus was shown using immunocytochemistry for the viral nucleoprotein after fixation, NP = green; nuclei = blue. (**D**) Cells were infected at 10^6^EID_50_ and supernatants were collected and titered for progeny virus production by standard plaque assay at indicated time points.

In our previous studies, rNA-wt showed two-fold higher virus titers than rSD20 on MDCK cells and replicated more effectively on chicken embryos. Meanwhile, *in vivo* experiments showed that rNA-wt and rSD20 respectively resulted in the greatest and lowest mortality in animals. Therefore, rNA-wt, the most virulent strain, and rSD20, the least virulent strain were designated for the proteomic study (Fig. 1A & B). To determine whether two H5N1 viruses could effectively replicate in CEF cells, cells were infected with both viruses at 10^6^EID_50_ and collected for detecting virus titers at 12, 24 and 36 hpi. Meanwhile, indirect immunofluorescence assay (IFA) for NP was carried out to verify H5N1 virus infection of CEF cells. Approximately, similar percents of CEF cells were infected by rNA-wt and rSD20 at 24 hpi with similar levels of cell pathologic effect (CPE) (Fig. 1C). At different times after infection, culture supernatants were harvested, serially diluted and assayed to determine the virus titer by using plaque assay. As shown in Fig. 1D, at 12, 24 and 36 hpi, the virus titers of rNA-wt were all higher than that of rSD20, which was in accordance with previous results in MDCK cells. Considering the severe CPE after 36 hpi, we therefore chose to study CEF cells at 12, 24 and 36 hpi to obtain cellular proteomic changes at different infection periods for 2-D experiments.

### DE proteins analysis

**Figure 2:**
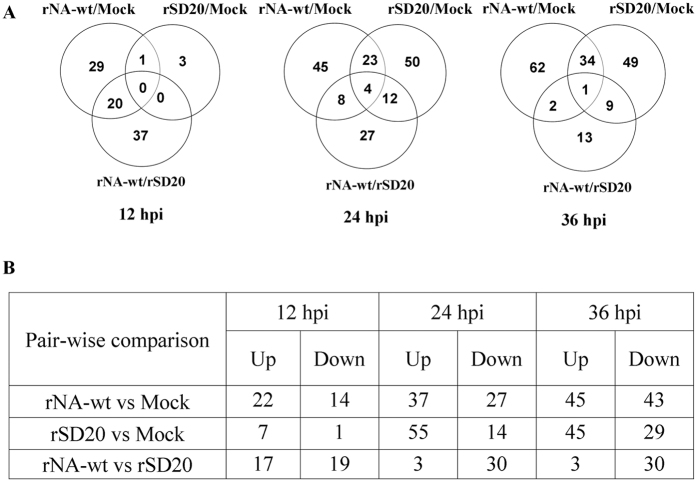
DE proteins in infected CEF cells by proteomic analysis. CEF cells were infected with the indicated viruses or with PBS and were collected at 12, 24 and 36 hpi for analysis. Based on image analysis by PDQuest software, pairwise comparisons of relative protein abundance were performed among the three groups (rNA-wt vs. control, rSD20 vs. control and rNA-wt vs. rSD20) and a number of significantly DE proteins as defined by >1.5-fold change in each group. The data represent the results from three independent infections for each virus. The numbers of proteins specific to individual group or shared by the two or three groups are represented in the Venn diagram (**A**), and the total numbers of DE proteins in the three groups are tabulated (**B**). The numbers of up- or down-regulated proteins in each group were also shown in the table (**B**).

Extracted total proteins from the CEF cells at different post infection times were prepared and loaded on to 2-DE gels. After the electrophoresis separation, the gels were stained with silver for assessing protein expression in CEF cells infected with two H5N1 viruses and control PBS at 12, 24 and 36 hpi. To compensate the variability of gel electrophoresis, three replicate gels were run for each time point. Based on image analysis by PDQuest software, pairwise comparisons of relative protein abundance were performed among the three groups (rNA-wt vs. control, rSD20 vs. control and rNA-wt vs. rSD20). The visual analysis of gels indicated total 441 DE protein spots across a pI range of 4–7 with at least 1.5-fold change among the three groups from each time point. At 12 hpi, only 80 spots differentially expressed in three groups and the rNA-wt infected cell (36 spots) displayed more spots than that of rSD20 (8 spots) when they were compared with the PBS-treated cells. However, at 24 and 36 hpi, rNA-wt and rSD20 showed similar DE protein spots. The protein spots in rNA-wt group were mostly downregulated and only 6 spots were up-regulated relative to that of rSD20 group. A representative master gel displaying proteins expressed in cells at each time point are illustrated in Fig. 2, and the locations of these DE protein spots were labeled with numbers.

### Protein identification and functional classification

Significantly differentially expressed proteins were identified by MALDI-TOF/TOF MS at 12, 24 and 36 hpi. Every protein spot was identified by at least two peptides. Identified proteins of 12, 24 and 36 hpi are listed in Supplementary Table S1-S3. In total, 47, 81 and 80 DE protein spots were identified at 12, 24 and 36 hpi respectively, although only some proteins identified from two or more different spots on the gel matched the same protein, such as vimentin. At 12 hpi, 19 proteins were significantly upregulated and 9 proteins were significantly downregulated in the rNA-wt infected group relative to the PBS control group, whereas only 2 proteins were significantly upregulated and 1 protein was significantly downregulated in the rSD20 virus infected group relative to the PBS control group. Meanwhile, 27 proteins were significantly upregulated and 9 proteins downregulated in the rNA-wt infected group relative to rSD20 infected group. Likewise, the DE spots at 24 and 36 hpi were also identified and displayed in Supplementary Fig. S1. Although some proteins, such as viral NP and NS1, vimentin, TGM2 and HSP90A, identified from two or three different spots on the gel matched the same protein in the NCBI nr database, the spots were clearly separated in the gels, suggesting that some regulated proteins had post-translational modification (PTM) or several kinds of cleavages. Forty-seven unique viral peptides from six specific proteins were detected exclusively during rNA-wt or rSD20 infection, but not in the control cells. According to annotations from UniProt knowledgebase (Swiss- Prot/TrEMBL) and Gene Ontology Database, the identified cellular proteins were classified into several categories based on their functional significance, including stress response, mRNA processing and splicing, transcription regulation, transport, metabolic process, cytoskeleton, molecular biosynthetic process, signal transduction, intermediate filament and apoptotic process. The detailed function information of DE proteins at each time point was shown in the Supplementary Data. The majority of the DE protein spots are illustrated in enlarged formats in the order of functional classification (Supplementary Fig. S2).

### Protein pathways analysis

**Figure 3:**
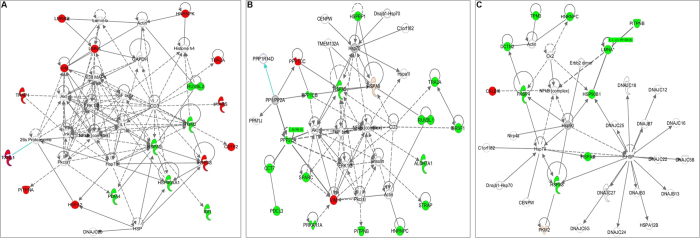
Protein networks significantly regulated (p < 0.05) by rNA-wt relative to rSD20 infection. DE proteins were analyzed by using Ingenuity Pathways Analysis (IPA, Ingenuity Systems) to determine cellular pathways impacted by rNA-wt relative to rSD20. (**A**) At 12 hpi, the functional network with the highest score was Post-Translational Modification, Protein Folding, DNA Replication, Recombination, and Repair with TGF-β at its center. The proteins in red and green were significantly up- or down-regulated expression (p < 0.05), respectively. Proteins in white are available in the IPA database but were not detected in this study. The shape of symbols denotes the molecular class of the proteins. A solid line indicates a direct molecular interaction, whereas a dashed line indicates an indirect molecular interaction. (**B**) At 24 hpi, the DE proteins of rNA-wt relative to rSD20 were mostly down-regulated and mainly involved in Post-Translational Modification, Cancer, Cardiovascular Disease, with TGF-β as central molecular. (**C**) At 36 hpi, the pathway of DE proteins of rNA-wt relative to rSD20 included Lipid Metabolism, Small Molecule Biochemistry, Vitamin and Mineral Metabolism.

DE proteins were analyzed using IPA to determine cellular pathways impacted by rNA-wt and rSD20 viruses. At 12 hpi, the regulated proteins of rNA-wt group compared with control were mainly involved in DNA Replication, Recombination and Repair, Energy Production, Cell Morphology, Cancer and Cellular Development. Due to only three DE proteins in rSD20 group, no significant pathways were obtained from the analysis results. However, after analyzing the DE proteins of rNA-wt relative to rSD20, it is interesting to find that most proteins are involved in the network of Post-Translational Modification, Protein Folding, DNA Replication, Recombination, and Repair with TGF-β as central molecular (Fig. 3A). At 24 hpi, the regulated proteins of rNA-wt group compared with control were mainly involved in Cellular Growth and Proliferation, Organ Morphology, Reproductive System Development and Function. In the group of rSD20 relative to control, the top pathways were cell death and survival, cellular growth and proliferation, dermatological diseases and conditions. Likewise, the DE proteins of rNA-wt relative to rSD20 which mostly were down-regulated mainly were involved in post-translational modification, cancer, cardiovascular disease, with TGF-β as central molecular (Fig. 3B). At 36 hpi, the pathway of DE proteins of rNA-wt relative to rSD20 included lipid metabolism, small molecule biochemistry, vitamin and mineral metabolism (Fig. 3C).

### rNA-wt induced more rapid response than rSD20

At 12 hpi, rNA-wt could replicate effectively in CEF cells and activate the host protein alteration. However, the attenuated rSD20 virus only induced three DE proteins (PURB, GDI2 and ATP5B). Compared with rSD20 group, most of the DE proteins (23/30) induced by rNA-wt were upregulated, which were mainly related to cytoskeleton (including intermediate filament), stress response, mRNA processing and splicing. In the identified cytoskeleton-associated proteins, CKAP4, ACTR1A, ACTG1, LMNB2, LMNA and vimentin were all upregulated with different levels of abundance. Alteration of cytoskeleton proteins might be necessary for influenza virus life cycle. Moreover, proteins with different levels of abundance involved in stress response and apoptosis were found in rNA-wt infected CEF cells. Five unique heat shock proteins (HSP75, HSP90A, HSPA2, HSPA5 and HSPA8) were identified during rNA-wt infection. HSP90A and HSPA5 were downregulated in rNA-wt group relative to rSD20 group. Besides, apoptosis-associated proteins, LMNA and HSPA5 were down-regulated in rNA-wt group relative to rSD20 group. During influenza virus infection, mRNA transcription and protein synthesis are maintained at high level and a dramatic switch from cellular to viral protein synthesis occurs despite the presence of high level of functional cellular mRNAs in the cytoplasm of infected cells. Therefore, it is not surprising that many regulated protein induced by rNA-wt were RNA processing and splicing (including TRA2A, ISY1 and CSTF2), transcription regulation (RUVBL2, PSPC1, HSPA8 and MRPL12) and protein biosynthesis (YARS, GARS and IDI1). Through IPA network analysis, these DE proteins participated in post-translational modification, protein folding and DNA replication, recombination and repair, with TGF-β as the central molecule (Fig. 3A).

### rSD20 produced a delayed host response

At 24 and 36 hpi, however, most DE proteins were up-regulated in rSD20 group compared to NA-wt, characterized by an increase of proteins involved in transcription regulation, mRNA processing and splicing, cytoskeleton, metabolic and stress response, indicating that rSD20 produced a delayed host response. Functional analysis of these proteins using IPA showed that the majority of the proteins were associated with host physiological functions, such as post-translational modification, cancer, lipid metabolism, and small molecule biochemistry (Fig. 3B & C). At first glance, this functional network illustrates the upregulation of a large proportion of proteins related to cell cycle regulation or metabolic process in rSD20 (PPP1CC, PPP1CB, PITPNB, ALDH7A1, ALDH6A1, PPP2CA and PKM2). This is interesting, because it has been shown that influenza virus infection induces cell cycle arrest, which promotes a favorable environment for influenza virus protein expression^16^. We also observed the upregulation of specific response components in rSD20, such as proteins associated with apoptosis (PDCL3, PPP2CA, PKM2, CTSD, LMNA, HSPA5 and HSP90B1) and proteins involved in stress response (HSPA8, HSPA2, HSPA5, IMMT and HSP90B1). This functional signature illustrates a striking dichotomy in the protein expression profiles during the course of the experimental infection; where infected cells elicited a steady upregulation of an acute-response environment that likely generated an antiviral state that partially protected the rNA-wt infected cells. Only four proteins, PPP1CC, vimentin, PKM2 and CALB1, in rSD20 group expressed lower than rNA-wt group. Network analysis of these proteins allowed us to identify a set of physically interacting proteins that might be responsible for survival.

### Strain-specific or unspecific DE proteins at three time points

Based on the rule that certain proteins were present in one virus group and absent in the other virus group, we selected several strain-specific DE proteins. At 12 hpi, vimentin, TRAP1, PITPNA, MRPL12, LMNA, HSPA8, HSP90AA1, HNRNPC, CKAP4, ACTR1A and A2M were specifically regulated in rNA-wt infected CEF cells. However, characteristically of rSD20 group, GDI2 and ATP5B were altered relative to control cell. Likewise, at 24 hpi, only PRDX4 was the specific DE protein in rNA-wt group, whereas, DE proteins, such as TPM3, PLS3, CCT5, DES, PITPNB and HSPA5 were regarded as the protein markers in rSD20 groups. Of these, TPM3, PLS3, CCT5 and DES were related to cytoskeleton. At 36 hpi, NPM1, EF1D and CHP as the regulators of I-κB kinase/NF-κB cascade, and RGN and ITPA, related to biosynthetic and catabolic process, were the specific molecules of rNA-wt group. Infection of rSD20 in CEF cells was characteristic of regulated LMNA, HSPA2 and FKBP4. Among the identified proteins, different products from the HSPs and Lamins gene family were found to be differentially expressed between NA-wt and rSD20 infected CEF cells relative to control cell at all time points. Meanwhile, only three proteins that displayed regulated expression in both H5N1-infected CEF cells at all the time points were ACTG1, LMNB2 and vimentin, which are cytoskeleton protein that is generally expressed in normal cells and reorganized after virus infection (Supplementary Data).

### Validation of the DE proteins by Western blot

**Figure 4:**
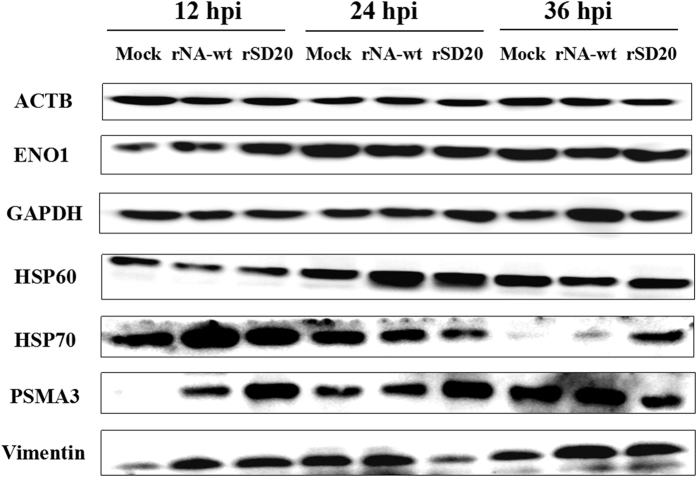
Western blot analysis of selected proteins. The proteins were detected from individual samples of CEF cells infected by rNA-wt, rSD20 or PBS that were collected at 12, 24 and 36 hpi. The inoculations are indicated at the top and the names of the six DE proteins, together with β-actin, are shown on the left. Full-length blots/gels are presented in Supplementary Figs S4~S10 respectively.

Western blot was performed to confirm the proteomic data for seven altered proteins at 12, 24 and 36 hpi in CEF cells infected with the two H5N1 viruses and control group. β-actin was often used as endogenous control in validating genomic or proteomic data, however, they have been reported upregulation in cells infected with influenza virus^17^. As shown in Fig. 4, no differences in the amount of β-actin were observed among the three different groups in protein level. However, GAPDH was regulated in virus infected CEF cells at certain times, indicating that it could not be chosen for endogenous control. At 12 hpi, the amount of HSP70 in the rNA-wt infected cells was greatly increased relative to rSD20 infected cells and control cells. At 24 and 36 hpi, it was increased highly in rSD20 infected CEF cells compared to rNA-wt infected cells and the control cells. Other proteins, like ENO1, HSP60 and PSMA3, were also consistent with the proteomic analyses, whereas vimentin was detected upregulated in rNA-wt and rSD20 infected CEF cells at 12 and 36 hpi, and largely reduced in rSD20 infected cells at 24 hpi. However, in the 2-DE results, vimentin was detected in the forms of both up- and down-regulation at three time points, which might be related to the post-translational modification. Therefore, quantitative real time polymerase chain reaction (qRT-PCR) was performed for the further confirmation.

### Validation of the DE proteins by qRT-PCR

**Figure 5:**
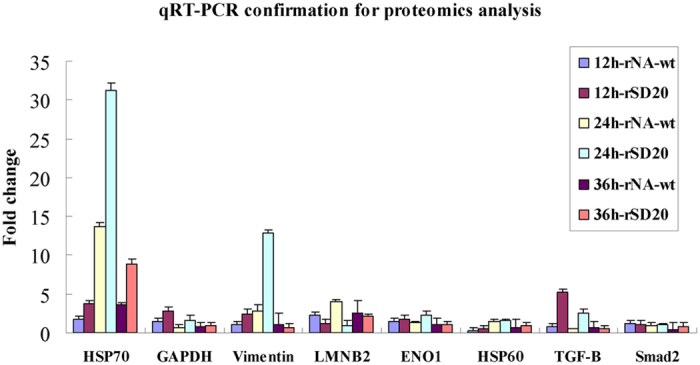
The mRNA levels of selected proteins analyzed by using qRT-PCR. Groups of CEF cells (n = 3) were infected with 10^6^ EID_50_ of the indicated virus or with PBS as a control. The cells were collected at 12, 24 and 36 hpi for mRNA extraction. qRT-PCR was performed to validate eight genes and the mRNA levels of selected genes at each time point were analyzed with chicken histone H5 gene as reference for expression normalization.

Based on the pathway networks stimulated by rNA-wt and rSD20 viruses, we chose TGF-β gene and Smad2 which participated in the TGF-β downstream signaling, and other regulated genes present in the top functional networks for qRT-PCR confirmation. Therefore, the following genes, vimentin, HSP70, HSP60, GAPDH, ENO1, LMNB2, TGF-β and smad2 were chose for qRT-PCR. As shown in Fig. 5, TGF-β in rSD20-infected cells were increased stably at 5.24 and 2.61 folds at 12 and 24 hpi respectively, relative to control group, whereas, it reduced at all the times in rNA-wt infected cells. The expression of Smad2 exhibited similar levels in the two viruses infected cells. The mRNA levels of vimentin, HSP70, HSP60, GAPDH, ENO1 and LMNB2 for the rNA-wt and rSD20 infected cells agreed with the proteomic data.

### DE proteins could regulate IFN-β response and affect virus replication

**Figure 6:**
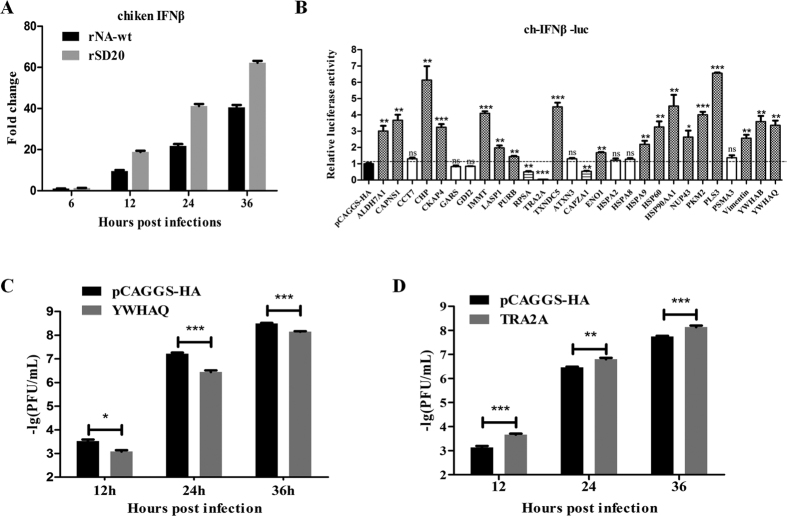
DE proteins modulate IFN-β production and affect virus replication. (**A**) H5N1 infection induces IFN-β gene expression in primary CEF cells. CEF cells were cultured and infected with H5N1 viruses, and then the total RNA extracted from the mock and virus infected cells was used for chicken IFN-β gene expression determination by qRT-PCR. The values were means ± SEs of three independent experiments. (**B**) The effect of DE proteins on IFN-β promoter activity induced by chicken derived MDA5. DF1 cells were transfected with 60 ng/well of pCAGGS-MDA5 (chicken), 400 ng/well of reporter plasmid (IFN-β-luc) and 10 ng/well of the pRL-TK as internal control, and 800 ng/well of indicated expression plasmids or an empty control plasmid. The cells were lysed 24 h later, and firefly luciferase and Renilla luciferase activities were determined with the Dual-Luciferase reporter assay system (Promega), according to the manufacturer’s protocol. (**C**,**D**) The effect of YWHAG and TRA2A proteins on virus replication. DF1 cells were transfected with 1 μg expression plasmid wihle transfected the pCAGGS-HA as a control group, and after 24 h, the cells were infected at a multiplicity of infection (MOI) of 0.01. At the indicated times postinfection, supernatant was collected and the viral titers were determined by plaque assay on MDCK cells.

Previous study showed influenza strains differ in their ability to induce type-I IFN, which correlated with the NA activity of these strains^18^. To evaluate the ability of the rNA-wt and rSD20 to induce IFN-β expression, IFN-β transcript was examined by qRT-PCR (Fig. 6A). The rSD20 appeared to induce the higher levels of IFN-β than the rNA-wt (p < 0.01). We detected the contribution of DE proteins to IFN-β promoter activity induced by chicken derived MDA5. Results showed that several DE proteins could significantly modulate IFN-β promoter activity induced by MDA5 when compared with that of pCAGGS-HA as control. Some of these proteins, such as ALDH7A1, CKAP4, CAPNS1, CHP, IMMT, TXNDC5, HSP90AA1, PKM2, PLS3, YWHAB and YWHAQ, strongly enhanced the IFN-β promoter activity. In contrast, TRA2A, RPSA and CAPZA1 significantly decreased the IFN-β promoter activity(Fig. 6B). We also explored the role of these DE proteins in H5N1 virus replication, and found that YWHAQ could inhibit viral replication(Fig. 6C), and TRA2A could enhance viral replication at all the time points(Fig. 6D).

### Confocal Microscopy

**Figure 7:**
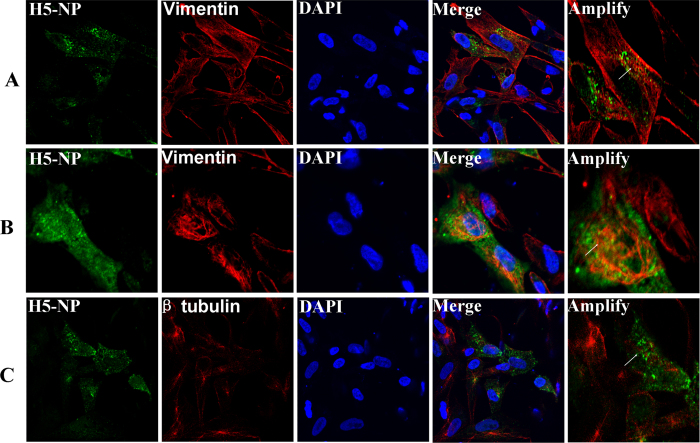
Subcellular distribution of cytoskeleton proteins vimentin and tubulin in H5N1 infected cells by IFA. NP protein was recognized with mice antiserum to H5N1-NP followed by FITC conjugated IgG (green). Vimentin and β-tubulin in H5N1 infected cells were detected using mouse mAbs followed by CY3-conjugated IgG (red). The nucleus was stained with DAPI (blue). The triple stained cells were observed by a Zeiss LSM510 laser confocal microscopy. The arrowheads represent uninfected cells, and arrows indicate H5N1 infected cells. (**A**) The subcellular location of vimentin filaments in H5N1 infected CEF cells using double staining IFA at 6 hpi. (**B**) The filamentous vimentin was considerably broken down in rNA-wt infected cells at 12 hpi. (**C**) The array of radial tubulin microtubules disappeared in the H5N1 infected CEF cells.

To visualize the cytoskeletal network in H5N1 virus-infected cells, an IFA was performed using the humanized mAbs to vimentin and β tubulin protein. Thus, we opted to examine the subcellular location change of vimentin filaments and β tubulin microtubules in rNA-wt -infected CEF cells using double staining IFA. When viral proteins were expressed at a low level at 6 hpi, the organization of vimentin displayed a steady distribution (Fig. 7A). At 12 hpi, it was obvious to find that viral NP protein localized at the areas of vimentin (Fig. 7B). As shown in Fig. 7C, the β tubulin was considerably broken down in rNA-wt infected cells at 12 hpi.

## Discussion

Avian H5N1 influenza virus is a highly contagious viral disease of major concern to the poultry industry and has also been recognized as a human health concern because of their potential to be episodically transmitted from poultry to human. In our previous study, the recombinant H5N1 viruses with stalkless-motif (rSD20) displayed an attenuated phenotype compared with parent virus (rNA-wt). The stalkless mutant rSD20 replicated to a titer slightly lower than that of the rNA-wt in cells and displayed a highest attenuation to mice and lower replication in mouse tissues. Moreover, rSD20 did not cause any visible disease or deaths in chickens. On the contrary, the rNA-wt caused the most deaths and highest viral shedding in chickens. The pathogenic differences might be related to differences in the tissue or cell tropism^2^,^4^, which has been previously shown to be influenced by the NA stalk lengths and could be related to an altered balance between hemagglutinin and neuraminidase activities^5^. Virus infection has multiple effects on host cell processes, such as cell signaling pathways and morphology. However, the difference of host cell response activated by H5N1 viruses with different NA stalk lengths remains unknown. High throughput quantitative proteomics is an ideal methodology to map such changes from the perspective of a total cell as it is suited to unbiased comparison analysis. In order to assess the effect of both strains (short-stalk and stalkless) and time points (12, 24, and 36 h) on protein expression, two-way ANOVA was performed using strains and time points as two independent variables. We identified 208 proteins that were differentially expressed, which were classified into stress response, mRNA processing and splicing, transcription regulation, transport, metabolic process, cytoskeleton, molecular biosynthetic process, signal transduction, intermediate filament and apoptotic process. Several representative DE proteins were confirmed by Western blot or qRT-PCR. It is demonstrated that the DE proteins between short-stalk and stalkless virus infection were mainly involved in TGF-β mediated signal networks. A number of the DE proteins identified in H5N1 infected CEF cells have been reported to play a role during certain stages of the infection process for a variety of viruses and this may provide a clue as to why they are regulated in the H5N1-infected CEF cells. These include:

### The innate immune responses of chicken cells against H5N1 virus

In mammals, innate immune system functions as the first line of host defense against viral infection. Pattern recognition receptors (PRRs) are a class of innate immune response expressed sensors that provide the host with the ability to detect and respond to virus infection, which then trigger a class of anti-viral signaling cascades, ultimately ensuring production of necessary effector molecules required for virus elimination^19^. In this context, retinoic acid-inducible gene-I (RIG-I) is the main cytosolic pattern-recognition receptor known for detecting influenza A virus infection in mammalian cells^20^. However, unlike mammals, chickens lack RIG-I, yet chicken cells produce type-I interferon (IFN) in response to AIV infection^21^. The avian defense mechanism against viral infection remains controversial. Some studies indicated that no IFN responses were observed in CEF cells infected with AIV^22^. In this study, although type-I IFN or effector molecules were not identified in the proteomic results, we found increased mRNA levels of IFN-β in infected CEF cells at each time point, suggesting the upregulation of IFN-β after H5N1 virus infections. It is a very striking to point that two 14-3-3 family proteins, YWHAB(14-3-3β) and YWHAQ(14-3-3θ), were upregulated in both viruses infected cells at 24 and 36 hpi. Through quantitative proteomics, it had been demonstrated that viral infection could activate 14-3-3 proteins in human keratinocytes and 14-3-3 proteins participate in the NF-κB signaling pathway^23^. Recently, Liu *et al*. further discovered that 14-3-3ε was essential for the stable interaction between RIG-I and TRIM25, which facilitated RIG-I ubiquitination and initiation of innate immunity against viruses, indicating a broad role for 14-3-3 family proteins in eukaryotic innate immunity^24^. Because of absence of RIG-I in chicken cell, in this study, upregulated YWHAB and YWHAQ may work by a different mechanism than it does in eukaryotic cells. Through overexpression experiments, we also found that both YWHAB and YWHAQS could enhance IFN-β promotor activity, and YWHAB displayed a significant antiviral potential against H5N1 virus. Several proteins that interact with 14-3-3ε, like HSP90AA1, RUVBL2, HNRPC, NPM1, EF1D, ATP5B and PP2CB, were identified, and they could participate in regulation of immune signal transduction with different ways^25^,^26^. Whether these proteins participate in regulating type-I IFN response remains to be elucidated. Besides, we identified several novel proteins that could modulate MDA5-medieated IFN-β promoter activity (Fig. 6B). For example, the expressions of ALDH7A1, HSP90AA1 and IMMT were higher in rSD20 infected cells than rNA-wt infected cells, while the expressions of TRA2A and CAPZA1 were lower in rSD20 infected cells than rNA-wt infected cells (Supplementary Table S1-S3). The IFN-β difference caused by the two viruses may be related with the specific DE proteins. How these proteins regulate type-I IFN response and viral replication remains to be elucidated. All of the above indicate that chicken may have a special innate immune mechanism against viral infection. Hence, although the exact molecular mechanisms and the pathways involved in NA-induced signaling remain unknown, direct and indirect events of the DE proteins identified in this study might be involved.

### The effect of NA stalk lengths on H5N1-activated TGF-β

Previous studies had showed that TGF-β could inhibit HCV and HIV replication and viral protein expression, and prevent pulmonary immunopathology in mice infected with influenza virus^27^,^28^,^29^,^30^. Influenza virus infection induces JNK-mediated TGF-β production in primary lung epithelial cells^31^. NA was shown to convert TGF-β from the latent to the active form to an extent sufficient to induce TGF-β dependent apoptosis^32^. However, it remains controversial which subtype of influenza virus could induce or activate TGF-β. On one hand, in an *in vitro* experiment, H1N1, but not HPAIV H5N1 virus, could induce a persistent elevation of TGF-β in human pulmonary epithelial cells^33^. On the other hand, *in vivo* experiments demonstrated that TGF-β was induced in mice lungs infected by non-HK (Hong Kong)-origin avian influenza viruses, but not induced by HK-origin HPAIV H5N1 viruses which resulted in more severe and widespread lesions^27^. Therefore, the severity of the HK-origin H5N1 infection suggests that decreased levels of TGF-β may be important in the pathogenesis. In our present study, through analyzing the DE proteins of rNA-wt relative to rSD20, it is interesting to find that most proteins are involved in the functional network mediated by TGF-β, especially at 24 hpi. Although as a secretary cytokine, TGF-β could not be detected in total proteins of CEF cells, its mRNA level was increased sharply in rSD20 infected group and reduced in rNA-wt infected group at 12 and 24 hpi (Fig. 5), indicating that rNA-wt virus might be unable to induce TGF-β expression compared with rSD20 virus. Generally, TGF-β activates two different signaling pathways mediated by Smads and TRAF6 respectively, leading to the activation of Smad2/3 and p38/JNK, which in turn results in the activation of transcription factors, such as p53 and pro-apoptotic gene expression^34^. Vimentin can be induced by TGF-β, and Smad2 is the main downstream molecular of TGF-β signaling, therefore Smad2 and vimentin were also selected for qRT-PCR confirmation. Results showed that Smad2 was similar in two viruses infected CEF cells and the expression level of vimentin was higher in rSD20 group than rNA-wt group at 12 and 24 hpi. In addition, several TGF-β regulated proteins reported previously, such as PPP2CA and HNRNPC, were lower in rNA-wt group than rSD20 group, indicating that rSD20 could activate TGF-β more effectively than rNA-wt in CEF cells^35^,^36^. Therefore, the NA of rNA-wt virus with a 20-amino-acid deletion in the stalk might be related to the decreased ability to induce or activate TGF-β compared with the rSD20 virus. Our studies suggest that neuraminidases with different stalk lengths may have different potential to directly regulate H5N1-induced TGF-β, which may in turn affect viral titers and disease outcome in animals and details on the mechanism deserves further research. In summary, the differential expression of TGF-β following infection with H5N1 influenza viruses with different NA stalk lengths might be a key determinant for diverse outcome.

### Cytoskeletal proteins in H5N1 virus infection

Viruses are obligate intracellular parasites and therefore depend on the host cellular machinery to facilitate entry, replication, transport, and release of progeny virions^37^. The intricate structural system referred to as the cytoskeletal network, necessary for viral infection, is composed of actin, microtubules, intermediate filaments, and motor proteins such as dynein and myosin^38^. It has been reported that many viruses interact with cytoskeletal elements, which is considered to be critical at each step along the replication cycle. In this study, when total DE proteins of these two H5N1 viruses infected CEF cells were performed by using IPA analysis, it was interesting to find that the most significant pathway were cell morphology, cell death and genetic disorder (Supplementary Fig. S3). As the main component of keeping cell morphology, many cytoskeletal proteins such as actin, cytoplasmic type 5 (ACTG1), intermediate filaments (vimentin, LMNA and LMNB2), actin-binding proteins (TPM3 TPM4 and PLS3), motor protein (dynein, kinesin and myosin), microfilament-associated proteins (SEPT2, SEPT11, CAPZA1 and CNN3) were identified at different time points. Surprisingly, as a major component of type III intermediate filaments, vimentin were found in the forms of up-regulated and down-regulated expressions. The subcellular localization of vimentin was investigated in rNA-wt-infected cells using confocal microscopy with β-tubulin as the control. The data indicated that the distribution of vimentin was predominately cytoplasmic in uninfected cells. However, in virus-infected cells, vimentin accumulated around the area of virus-infection and could co-localize with NP in the cytoplasm, indicating that virus infection induced vimentin reorganization. On the contrary, after virus infection, β-tubulin was collapsed and dispersed in virus infected areas, which indicated that H5N1 virus could manipulate and utilize different host cytoskeletal proteins to promote infection (Fig. 7). It has been previously reported that vimentin showed rearrangement from a radial pattern into an array surrounding the nucleus after influenza virus infection and was required for effective viral replication^39^. The unique roles of each cytoskeletal protein in the life cycle of H5N1 influenza virus remain unknown and worth future studies.

## Conclusions

In conclusion, we have profiled proteome changes in the CEF cells during various infection stages of two H5N1 viruses with different NA stalk lengths. Our findings indicate that different NA stalk lengths may play a significant role in various cellular processes. This study on these processes induced by these two viruses will enhance the understanding on the underlying mechanisms of genetic regulation of resistance to H5N1 virus with different NA stalk lengths.

## Methods

### Cell cultivation, virus infection and protein extraction

CEF cells were isolated from 10-day chicken embryos and grown in DMEM medium supplemented with 10% FBS (Gibco, Karlsruhe, Germany). The H5N1 viruses, rNA-wt (HA and NA genes from H5N1 virus, other genes from the A/WSN/33 virus) and rSD20 (a 20-amino-acid deletion in the NA stalk) were produced by reverse genetics as described previously^2^, cultivated in 11-day chicken embryos and reserved at −80 °C. Cells grown in T-flasks (75 cm^2^) were infected with two H5N1 viruses at 10^6^EID_50_. As untreated control, a mock-infection was performed, simulating the procedure for virus infection without virus addition. Titration of influenza virus was determined by plaque assay (PFU) and expressed as -lgPFU/ml. Proteins were respectively extracted at 12, 24, and 36 hours post-infection (hpi). At each time point, the cells of each group were washed twice with PBS, transferred to sterile tubes containing 500 μL of lysis buffer. The mixture was disrupted by sonication, incubated on ice for one hour with vortexing every 15 minutes and finally centrifuged at 12,000 × g for 30 minutes at 4 °C. The supernatants were aliquoted and stored in −80 °C freezer before the following 2-DE. The protein concentration was determined using the Plus One 2-D Quant Kit (GE Healthcare, USA). All works with live H5N1 viruses were conducted in the BSL-3 facilities at Huazhong Agricultural University.

### 2-D electrophoresis

IEF was performed using the IPG phor II system (GE Healthcare) and Immobiline 24-cm DryStrip IPG strips (pH 4–7). The prepared proteins (150 mg/strip) were mixed with rehydration buffer (7 M urea, 2 M thiourea, 2% w/v CHAPS, 1% w/v DTT, and 0.5% v/v IPG buffer, pH 4–7, with 0.002% w/v bromophenol blue). The protein samples were focused for a total of 70 kV∙h. After IEF, the IPG strips were equilibrated with 10 mg/mL of DTT and 40 mg/mL of iodoacetamide for 15 minutes in equilibration buffer (2% w/v SDS, 30% v/v glycerol, 0.002% w/v bromophenol blue, 6 M urea, and 50 mM Tris-HCl; pH 8.8). The 2-D electrophoresis was then performed on a 10% SDS-PAGE using an EttanTM DALTSix electrophoresis unit (GE Healthcare).

### Image scanning and analysis

The proteins were visualized by silver staining, and image analyses were performed using the Image Master V6.01 program (GE Healthcare). Before the image analysis, normalization of background was performed to adjust for differences in protein spot intensity. Biological variation analysis was performed to investigate the DE protein spots among the different sample groups after differential in-gel analysis was carried out in each gel. The gel with the most detected protein spots was chosen as the master image, against which the spots of all of the other gel images were matched. DE protein spots with a change greater than 1.5-fold and a p-value of less than 0.05 (Student’s t-test) between groups were considered as up- or down-regulated and chosen for MS identification.

### Protein identification by MS/MS

The DE protein spots were excised from the silver-stained gels and transferred to V-bottom 96-well microplates loaded with 100 μL of 50% ACN/25 mM ammonium bicarbonate solution per well. After destaining for 1 hour, the gel plugs were dehydrated with 100 μL of 100% ACN for 20 minutes and then completely dried in a SpeedVac concentrator (Thermo Savant) for 30 minutes. The dried gel particles were rehydrated at 4 °C for 45 minutes with 2 μL/well of trypsin (Promega) in 25 mM ammonium bicarbonate and then incubated at 37 °C for 12 hours. After trypsin digestion, the peptide mixtures were extracted with 8 μL of extraction solution (50% ACN/0.5% TFA) per well at 37 °C for 1 hour prior to MALDI-TOF MS/MS analysis. Matrix was prepared by dissolving α-cyano-4-hydroxycinnamic acid (Bruker daltonics, Breman, Germany) in 50% ACN and 0.1% TFA to make a 5 mg/ml solution. It was sonicated for 15 min and centrifuged at 10,000 rpm for 5 min. Mass spectra were obtained on an Ultraflex III MALDI TOF/TOF mass spectrometer (ABI4700, USA), positive mode in the mass range of 700–3200 Da with an accelerating voltage of 20 kV. The Nd: YAG laser was operated at a wavelength of 355 nm. Two microliters of peptides mixture was mixed with equal amount of matrix clear supernatant and 0.5 μl of this was spotted on MALDI ground steel target plate. Peptide ion masses were internally calibrated using trypsin autolytic peptides at m/z 702.366 and 3419.542. MALDI analysis was performed by a fuzzy logic feedback control system (Ultraflex αMALDI TOF/TOF system Bruker, Germany) equipped 8 with delayed ion extraction, applying the same peak detection and annotation parameters for both preparations. Parent mass peaks with mass range of 700–3200 Da and minimum signal to noise ratio of 50 were picked out for tandem TOF/TOF analysis. Protein identification was performed by searching extracted peak lists generated with one defined set of filter parameters against the NCBI database using the Mascot (Matrix Science, London, UK) search engine (http://www.matrixscience.com). The parameters and conditions were set as the following: tryptic digestion, maximum 2 missed cleavages, carbamidomethylation of cysteine as the fixed modification, oxidation of methionine as the variable modification, peptide mass tolerance of 50 ppm, fragment MS tolerance of 0.2 Da. MASCOT protein scores (based on combined MS and MS/MS spectra) of greater than 72 were considered statistically significant (p < 0.05).

### Bioinformatics Analysis

The functional classification and cellular localization of the identified cellular proteins was performed using the UniProt Knowledgebase (Swiss-Prot/TrEMBL). For pathway analysis, human NCBI gene identifiers corresponding to 208 of the differentially expressed chicken proteins were imported into the Ingenuity Pathways Analysis (Redwood City, CA) and UniProt knowledgebase programs. Each gene identifier was mapped to its corresponding gene object in the Ingenuity Pathways Knowledge Base. A cutoff of 2.0 was set to identify genes whose expression was significantly differentially regulated. Differentially expressed proteins within significantly altered pathways were consolidated into an interconnected network. Networks of these focus genes were then algorithmically generated based on their connectivity.

### Western blot

Mock- and influenza-infected CEF cells were scraped into cold PBS at 12, 24 and 36 hpi, pelleted at 5000 × g for 5 min, and lysed with cell lysis buffer (Kangwei, China). Forty micrograms of protein was loaded per lane into SDS-PAGE gels, separated, and transferred to nitrocellulose membranes. After blocking with 0.5% BSA, membranes were inoculated with primary antibodies for one hour using human ENO1 (sc-100812) form Santa Cruz, PSMA3 (Catalog# 3766-1), vimentin (Catalog# 2707-1), HSP90 (Catalog# 3670-1), HSP60 (Catalog# 1777-1), from Epitomics, HSP70 (#SPA-810) from Stressgen (Victoria, BC, Canada), and GAPDH (Catalog# 2251-1) and β-Actin from Sigma. The blots were then separately incubated with corresponding HRP-conjugated anti-mouse IgG and HRP-conjugated anti-rabbit IgG (KPL, USA) for another hour. After washing for three times, bands were detected with ECL (Thermo, USA) by DNR Bio-Imaging System.

### qRT-PCR

Proteomic results were validated by qRT-PCR using SYBR green-based detection with an ABI PRISM 7500 cycler (Applied Biosystems, Foster City, CA). The CEF cells monolayers inoculated with two H5N1 viruses for 12, 24, and 36 h were washed with ice-cold PBS and then lysed with TRIzol reagent (Invitrogen, USA). Total cellular RNA was extracted using the RNeasy minikit (Qiagen, GmbH, Hilden, Germany) according to the manufacturer’s protocol. After DNA inactivation, 2 μg RNA was reversely transcribed in a 20 uL reaction mixture containing 2 uL avian myeloblastosis virus (AMV) buffer, 50 pM Oligo18T, 0.5 mM dNTPs, 10 U RNase inhibitor and 20 U AMV reverse transcriptase (TAKARA, Japan). After initial denaturation at 95 °C for 30 s, the amplification was carried out through 40 cycles, each consisting of denaturation at 95 °C for 15 s, primer annealing at 60 °C for 30 s, and DNA extension at 72 °C for 45 s. Melting curves were obtained, and quantitative analysis of the data was performed using the 7500 System SDS software Version 1.3.1 in a relative quantification (ddCt) study model (Applied Biosystems). Primers were designed using Primer Premier 5.0 software based on chicken gene sequences in GenBank (Supplementary Table S4). The chicken histone H5 gene was used for expression normalization, using the primers described previously^40^. Changes in gene expression revealed by qRT-PCR were calculated by following the t-test reported previously^41^, and a P-value < 0.05 was considered significant.

### Luciferase reporter assays

To detect the contribution of DE proteins to IFN-β promoter activity induced by chicken derived MDA5, DF1 cells were transfected with 60 ng/well of pCAGGS-MDA5 (chicken), 400 ng/well of reporter plasmid (IFN-β-luc) and 10 ng/well of the pRL-TK as internal control, and 800 ng/well of indicated expression plasmids or an empty control plasmid. The cells were lysed 24 h later, and firefly luciferase and Renilla luciferase activities were determined with the Dual-Luciferase reporter assay system (Promega), according to the manufacturer’s protocol. In order to clearly show the difference between the chicken genes group and the control group, data were compared to a set control value (the pCAGGS-HA group value is set to “1”)

### Virus growth *in vitro*

To determine the effect of DE proteins on growth of the virus *in vitro*, DF1 cells were transfected with 1 μg indicated plasmids, and after 24 h, the cells infected at a multiplicity of infection (MOI) of 0.01 for 1 h and incubated at 37 °C in DMEM containing 0.7 μg/ml of tosylsulfonyl phenylalanyl chloromethyl ketone (TPCK)-treated trypsin. At the indicated times postinfection, supernatant was collected and stored at −80 °C. Viral titers were determined by plaque assay on MDCK cells.

### Confocal Microscopy

For indirect immunofluorescence staining, CEF cells were grown on coverslips. After 12, 24 and 36 hpi, cells were fixed with 4% paraformaldehyde and permeabilized with ice-cold acetone for 5 min. Cells were treated with a mixture of mAb H5N1-NP prepared in our laboratory and other host protein antibody for 1 h at 37 °C in a humidified chamber followed by treatment with secondary, species-specific Abs conjugated with FITC or Cy3 (Molecular Probes) for 1 h. Coverslips containing fixed samples were mounted onto slides using Vectorshield containing DAPI (Vector Laboratories) to allow visualization of cell nuclei. Confocal images were captured on a LSM510 META microscope (Carl Zeiss Ltd.) equipped with 40-fold, numerical aperture 1.4, oil immersion lenses. Pinholes were set to allow optical sections of 1 nm to be acquired. The fluorescence was measured in the linear range as the detector was a photomultiplier, and the range indicator was utilized to ensure that no saturated pixels were obtained on image capture. Images were averaged three times.

## Additional Information

**How to cite this article**: Li, Y. *et al*. Proteome Response of Chicken Embryo Fibroblast Cells to Recombinant H5N1 Avian Influenza Viruses with Different Neuraminidase Stalk Lengths. *Sci. Rep.* **7**, 40698; doi: 10.1038/srep40698 (2017).

**Publisher's note:** Springer Nature remains neutral with regard to jurisdictional claims in published maps and institutional affiliations.

## Supplementary Material

Supplementary Information

Supplementary Data S1
