# Supplementary material for: Proteome Response of Chicken Embryo Fibroblast Cells to Recombinant H5N1 Avian Influenza Viruses with Different Neuraminidase Stalk Lengths
Source: Sci Rep. 2017 Jan 12;7:40698. doi: 10.1038/srep40698 (PMC5227709; doi:10.1038/srep40698)

**Proteome Response of Chicken Embryo Fibroblast Cells to H5N1 Avian Influenza Viruses with Different Neuraminidase Stalk Lengths**

Yongtao Li, Fan Ming, Huimin Huang, Kelei Guo, Huanchun Chen, Meilin Jin, Hongbo Zhou

Supplementary materials

**Supplementary Table S1. Pairwise comparison of DE proteins in CEF at 12 hpi with the H5N1 virus rNA-wt or rSD20.**

| **Spot**  **ID^a^** | **Protein name**  **(Abbreviation)** | **Accession**  **No^b^** | **Differentially expressed proteins identified in CEF between groups** | | | | | | **Protein**  **score^d^** | **Matched peptide^e^** |
| --- | --- | --- | --- | --- | --- | --- | --- | --- | --- | --- |
|  |  |  | **rNA-wt and Mock** | | **rSD20 and Mock** | | **rNA-wt and rSD20** | |  |  |
|  |  |  | ***P-*value** | **Ratio^c^** | ***P-*value** | **Ratio** | ***P-*value** | **Ratio** |  |  |
| 1-01 | HSP90AA1 | gi|157954047 | 7.2e-40 | -2.98952 | - | -^f^ | 7.2e-40 | -2.98734 | 453 | 14 |
| 1-02 | HSP90AA1 | gi|326921046 | 1.8e-33 | -5.34 | - | - | 1.8e-33 | -5.215 | 389 | 12 |
| 1-03 | TGM2 | gi|62903517 | 9.1e-15 | -2.12892 | - | - | 9.1e-15 | -1.96899 | 202 | 8 |
| 1-04 | TGM2 | gi|62903517 | 4.6e-07 | -2.59978 | - | - | 4.6e-07 | -2.69666 | 125 | 11 |
| 1-06 | PDIA4 | gi|57530768 | 1.4e-13 | -2.3599 | - | - | 1.4e-13 | -3.96025 | 190 | 11 |
| 1-08 | Vimentin | gi|114326309 | 1.4e-29 | -4.40576 | - | - | - | - | 350 | 11 |
| 1-10 | PURB | gi|74228215 | 0.00021 | -2.1113 | 0.00021 | -2.201 | - | - | 98 | 2 |
| 1-11 | HNRPC | gi|119586801 | 0.0044 | -100^g^ | - | - | - | - | 85 | 2 |
| 1-13 | ISY1 | gi|50754283 | 2.3e-28 | -4.97622 | - | - | 2.3e-28 | 3.66532 | 338 | 8 |
| 1-14 | IDI1 | gi|118085591 | 9.1e-13 | -100 | - | - | 9.1e-13 | -26.9601 | 182 | 5 |
| 2-06 | PLS3 | gi|57530180 | 1.4e-26 | 3.75857 | - | - | 1.4e-26 | 3.17686 | 320 | 11 |
| 2-07 | CSTF2 | gi|224097289 | 0.0017 | 4.83451 | - | - | 0.0017 | 3.59968 | 89 | 5 |
| 2-09 | PSPC1 | gi|71895115 | 2.9e-19 | 2.04145 | - | - | 2.9e-19 | 3.59974 | 247 | 11 |
| 2-11 | CKAP4 | gi|118082813 | 3.6e-17 | 3.02347 | - | - | - | - | 226 | 10 |
| 2-12 | nucleoprotein (NP) | gi|122920718 | 9.6e-14 | 3.69719 | - | - | 9.6e-14 | 100 | 202 | 7 |
| 2-13 | CCT7 | gi|71895883 | 0.0059 | 3.06594 | - | - | 0.0059 | 3.20588 | 84 | 6 |
| 2-14 | LRFN5 | gi|189530677 | 0.012 | 100 | - | - | 0.012 | 4.23754 | 76 | 5 |
| 2-15 | nucleoprotein (NP) | gi|94483633 | 7.6e-14 | 3.22573 | - | - | 7.6e-14 | 1.91797 | 203 | 8 |
| 2-17 | CALU | gi|47498076 | 3.6e-06 | 2.96307 | - | - | - | - | 116 | 5 |
| 2-22 | PITPNB | gi|86129444 | 2.3e-17 | 3.69934 | - | - | - | - | 228 | 6 |
| 2-23 | LMNA | gi|45384214 | 9.1e-14 | 100 | - | - | 9.1e-14 | 100 | 192 | 16 |
| 2-24 | HSP75 | gi|57525126 | 4.6e-17 | 1.8571 | - | - | 4.6e-17 | 100 | 225 | 10 |
| 2-26 | LMNB2 | gi|45384202 | 2.3e-09 | 1.70115 | - | - | 2.3e-09 | 1.88630 | 148 | 14 |
| 2-27 | PSPC1 | gi|71895115 | 2.3e-18 | 1.71414 | - | - | 2.3e-18 | 3.14029 | 238 | 5 |
| 2-30 | SEPT2 | gi|57525156 | 5.8e-30 | 1.99755 | - | - | - | - | 354 | 8 |
| 2-31 | TPM4 | gi|515694 | 7.2e-11 | 1.67071 | - | - | - | - | 163 | 4 |
| 2-32 | LASP1 | gi|293601661 | 1.4e-11 | 1.81408 | - | - | - | - | 170 | 6 |
| 2-33 | SRSF1 | gi|82233969 | 5.8e-31 | 1.58827 | - | - | - | - | 364 | 10 |
| 2-34 | TXNL1 | gi|50806608 | 2.3e-15 | 1.73398 | - | - | 2.3e-15 | 1.95991 | 208 | 6 |
| 4-08 | GDI2 | gi|45384364 | - | - | - | - | - | - | 109 | 8 |
| 4-09 | ATP5B | gi|71897237 | - | - | - | - | - | - | 498 | 10 |
| 5-02 | A2M | gi|157954061 | - | - | - | - | 4.5e-19 | 100 | 245 | 7 |
| 5-05 | Vimentin | gi|114326309 | - | - | - | - | 7.2e-25 | 100 | 303 | 8 |
| 5-12 | HNRNPK | gi|50762370 | - | - | - | - | 9.1e-24 | 2.99769 | 292 | 14 |
| 5-14 | ACTR1A | gi|56118984 | - | - | - | - | 1.4e-12 | 100 | 180 | 4 |
| 5-15 | TRA2A | gi|57530708 | - | - | - | - | 0.01 | 3.23497 | 81 | 3 |
| 5-17 | ACTG1 | gi|56119084 | - | - | - | - | 7.2e-12 | 3.10598 | 173 | 6 |
| 5-21 | GARS | gi|71895709 | - | - | - | - | 1.4e-11 | 2.08111 | 170 | 13 |
| 5-22 | HSPA2 | gi|55742654 | - | - | - | - | 0.00024 | 12.8977 | 98 | 6 |
| 5-23 | HSPA8 | gi|6729733 | - | - | - | - | 5.8e-10 | 100 | 154 | 10 |
| 5-29 | YARS | gi|57530465 | - | - | - | - | 4.5e-16 | 2.17022 | 215 | 12 |
| 5-31 | nucleoprotein (NP) | gi|440246 | - | - | - | - | 4.8e-23 | 1.64771 | 295 | 9 |
| 5-37 | SEPT1 | gi|118090250 | - | - | - | - | 1.1e-18 | 4.48945 | 241 | 5 |
| 5-39 | PITPNA | gi|326931348 | - | - | - | - | 5.7e-35 | 100 | 404 | 8 |
| 6-08 | HSPA5 | gi|45382769 | - | - | - | - | 4.6e-27 | -2.70947 | 325 | 12 |
| 6-13 | RUVBL2 | gi|148230609 | - | - | - | - | 7.2e-05 | -2.12725 | 103 | 10 |
| 6-16 | GAPDH | gi|63401 | - | - | - | - | 3.6e-13 | -2.47182 | 186 | 4 |
| 6-19 | MRPL12 | gi|118099877 | - | - | - | - | 1.8e-17 | -100 | 229 | 5 |

**^a^** Spot ID represents the number on the representative gel in Fig. 2.

**^b^** Accession number is the MASCOT result of a MALDI-TOF/TOF search of the NCBInr database.

**^c^** Ratio stands for the average protein abundance ratio for different groups. A positive value indicates that protein expression

was upregulated in the first group; a negative value indicates that protein expression was downregulated in the first group.

**^d^** Protein scores (based on combined MS and MS/MS spectra) were derived from MALDI-TOF/TOF identification. Proteins with a statistically significant protein score of great than 72 were considered successfully identified.

**^e^** The number of peptides identified by using MS/MS is given by MASCOT.

**^f^** The symbol “-” means the p-value of the t-test is >0.05 or the Ratio is <1.5.

**^g^** The ratio of 100 indicates that the protein spots were newly induced (100) or absent (-100) in one virus-infected CEF relative

to the other virus infected CEF or mock CEF.

**Supplementary Table S2. Pairwise comparison of DE proteins in CEF at 24 hpi with the H5N1 virus rNA-wt or rSD20.**

| **Spot**  **ID^a^** | **Protein name (Abbreviation)** | **Accession**  **No^b^** | **Differentially expressed proteins identified in CEF between groups** | | | | | | **Protein**  **score^d^** | **Matched peptide^e^** |
| --- | --- | --- | --- | --- | --- | --- | --- | --- | --- | --- |
|  |  |  | **rNA-wt and Mock** | | **rSD20 and Mock** | | **rNA-wt and rSD20** | |  |  |
|  |  |  | ***P-*value** | **Ratio^c^** | ***P-*value** | **Ratio** | ***P-*value** | **Ratio** |  |  |
| 13-02 | TPM3 | gi|53129586 | 1.4e-11 | -1.75269 | -^f^ | - | - | - | 170 | 10 |
| 13-03 | ACTG1 | gi|56119084 | 4.5e-17 | -4.71899 | - | - | - | - | 225 | 7 |
| 13-06 | Vimentin | gi|114326309 | 1.4e-18 | -2.90254 | 1.4e-18 | -1.67038 | - | - | 240 | 11 |
| 13-07 | PKM2 | gi|45382651 | 1.3 | -1.88687 | - | - | - | - | 80 | 11 |
| 13-09 | CKAP4 | gi|118082813 | 3.6e-17 | -3.02347 | - | - | 3.6e-17 | -1.53791 | 226 | 10 |
| 13-13 | CKAP4 | gi|118082813 | 5.8e-15 | -3.49723 | 5.8e-15 | -1.87667 | - | - | 204 | 8 |
| 13-15 | CALU | gi|47498076 | 3.6e-06 | -4.10483 | 3.6e-06 | -2.34856 | - | - | 116 | 5 |
| 13-16 | PURB | gi|74228215 | 0.00021 | -3.40835 | - | - | - | - | 98 | 2 |
| 13-18 | ATXN3 | gi|45383440 | 1.8e-09 | -1.66241 | - | - | - | - | 149 | 7 |
| 13-19 | WDR77 | gi|71895697 | 1.8e-34 | -1.50241 | - | - | - | - | 399 | 8 |
| 13-20 | RPSA | gi|308081909 | 9.1e-36 | -11.7955 | 9.1e-36 | -9.48902 | - | - | 412 | 8 |
| 13-21 | SPARC | gi|45383337 | 5.8e-16 | -4.96284 | 5.8e-16 | -3.01169 | 5.8e-1 | -1.64786 | 214 | 7 |
| 13-22 | CTSD | gi|45384002 | 1.8e-05 | -1.68353 | - | - | - | - | 109 | 2 |
| 13-23 | HNRNPA3 | gi|118093536 | 0.00017 | -1.7186 | - | - | - | - | 99 | 8 |
| 13-24 | AKR1D1 | gi|118082901 | 0.00011 | -1.62081 | - | - | - | - | 101 | 6 |
| 13-26 | HNRNPC | gi|119586801 | 0.00017 | -4.05883 | 0.00017 | -100^g^ | - | - | 99 | 3 |
| 13-27 | ANXA8 | gi|50749462 | 0.00036 | -1.64516 | - | - | - | - | 96 | 3 |
| 13-28 | TRA2A | gi|57530708 | 5.7e-10 | -2.22914 | - | - | - | - | 154 | 4 |
| 13-29 | SRSF1 | gi|82233969 | 5.8e-31 | -2.57722 | - | - | 5.8e-31 | -3.20981 | 364 | 10 |
| 13-30 | TXNL1 | gi|50806608 | 2.3e-15 | -2.33465 | - | - | - | - | 208 | 6 |
| 14-04 | HSPA8 | gi|225698069 | 4.5e-11 | 3.61607 | 4.5e-11 | 2.46501 | - | - | 165 | 8 |
| 14-05 | UBQLN1 | gi|118104137 | 2.3e-25 | 2.8849 | - | - | - | - | 308 | 8 |
| 14-06 | HSPA9 | gi|57524986 | 2.9e-07 | 2.17375 | - | - | - | - | 127 | 9 |
| 14-07 | CSTF2 | gi|224097289 | 0.0017 | 3.51423 | 0.0017 | 5.85407 | - | - | 89 | 5 |
| 14-08 | DPYSL2 | gi|45383177 | 0.0012 | 1.91438 | 0.0012 | 2.53481 | - | - | 91 | 7 |
| 14-10 | PSPC1 | gi|71895115 | 2.9e-19 | 1.86149 | - | - | - | - | 247 | 11 |
| 14-13 | ACTB | gi|297302291 | 5.8e-06 | 2.77377 | 5.8e-06 | 2.60071 | - | - | 114 | 4 |
| 14-14 | nucleoprotein (NP) | gi|94483633 | 7.6e-20 | 2.2654 | 7.6e-20 | 1.88030 | - | - | 263 | 7 |
| 14-16 | nucleoprotein (NP) | gi|94483633 | 7.6e-14 | 1.61553 | 7.6e-1 | 1.66682 | - | - | 203 | 8 |
| 14-17 | FKBP4 | gi|57525441 | 7.2e-0 | 100 | 7.2e-06 | 100 | - | - | 113 | 6 |
| 14-20 | TXNDC5 | gi|57530789 | 1.1e-22 | 1.75177 | 1.1e-22 | 2.0616 | - | - | 281 | 6 |
| 14-21 | Vimentin | gi|114326309 | 5.8e-32 | 3.63527 | 5.8e-32 | 2.69465 | - | - | 374 | 15 |
| 14-23 | ACTG1 | gi|56119084 | 9.1e-18 | 100 | 9.1e-18 | 100 | - | - | 232 | 5 |
| 14-25 | PPP1CC | gi|57525187 | 2.9e-19 | 6.59573 | 2.9e-19 | 3.14431 | - | - | 247 | 12 |
| 14-26 | Vimentin | gi|57240090 | 3.6e-37 | 3.56563 | 3.6e-37 | 2.87104 | - | - | 426 | 15 |
| 14-27 | Vimentin | gi|57240090 | 7.2e-20 | 3.02113 | 7.2e-20 | 2.43135 | - | - | 253 | 10 |
| 14-28 | YWHAQ | gi|55741594 | 9.1e-25 | 2.4181 | 9.1e-25 | 1.91324 | - | - | 302 | 14 |
| 14-29 | YWHAB | gi|57529350 | 9.1e-08 | 2.03045 | - | - | - | - | 132 | 7 |
| 14-30 | PRDX4 | gi|118084001 | 4.5e-22 | 100 | - | - | - | - | 275 | 9 |
| 14-31 | LMNB2 | gi|45384202 | 9.1e-16 | 3.99084 | - | - | - | - | 212 | 11 |
| 14-32 | PRDX4 | gi|118084001 | 1.1e-29 | 100 | - | - | - | - | 351 | 12 |
| 14-33 | Influenza NS1 | gi|262400876 | 4.8e-14 | 100 | 4.8e-14 | 20.3303 | 4.8e-14 | 1.58622 | 205 | 6 |
| 14-36 | Vimentin | gi|57240089 | 3.6e-19 | 4.1472 | 3.6e-19 | 2.48416 | 3.6e-19 | 1.66946 | 246 | 8 |
| 14-39 | MSN | gi|50513540 | 0.0013 | 1.47416 | 0.0013 | 1.81863 | - | - | 90 | 8 |
| 15-12 | TPM3 | gi|53129586 | - | - | 9.1e-15 | -100 | - | - | 202 | 13 |
| 15-13 | CAPNS1 | gi|2506056 | - | - | 1.1e-07 | -2.03858 | - | - | 131 | 3 |
| 16-07 | LMNA | gi|45384214 | - | - | 2.9e-25 | 1.81983 | - | - | 307 | 22 |
| 16-10 | SDHA | gi|3851616 | - | - | 9.1e-07 | 1.60377 | - | - | 122 | 7 |
| 16-14 | HSPA8 | gi|190576828 | - | - | 0.04 | 1.86482 | 0.04 | -1.69484 | 76 | 6 |
| 16-15 | PLS3 | gi|57530180 | - | - | 1.4e-26 | 100 | - | - | 320 | 11 |
| 16-16 | LAMB2 | gi|45384202 | - | - | 9.1e-11 | 1.9188 | 9.1e-11 | -1.95321 | 162 | 13 |
| 16-20 | HSPA5 | gi|45382769 | - | - | 7.2e-06 | 100 | 7.2e-06 | -7.31057 | 113 | 7 |
| 16-21 | KLC4 | gi|118087579 | - | - | 0.0018 | 3.26974 | - | - | 89 | 5 |
| 16-22 | HSPD1 | gi|61098372 | - | - | 1.1e-10 | 3.5917 | - | - | 161 | 4 |
| 16-24 | CCT7 | gi|71895883 | - | - | 7.2e-29 | 1.66445 | - | - | 343 | 14 |
| 16-25 | PKM2 | gi|45382651 | - | - | 5.7e-07 | 1.53592 | - | - | 124 | 7 |
| 16-26 | CCT5 | gi|60302774 | - | - | 1.8e-14 | 100 | - | - | 199 | 9 |
| 16-27 | Desmin(DES) | gi|2959450 | - | - | 7.2e-40 | 100 | - | - | 453 | 16 |
| 16-28 | TXNL1 | gi|50806608 | - | - | 1.4e-08 | 1.93387 | - | - | 140 | 7 |
| 16-33 | CCT2 | gi|60302718 | - | - | 4.6e-15 | 1.88524 | - | - | 205 | 8 |
| 16-37 | RUVBL2 | gi|89266849 | - | - | 1.8e-19 | 4.01171 | - | - | 249 | 12 |
| 16-39 | GRSF1 | gi|118090272 | - | - | 5.7e-25 | 2.36094 | - | - | 304 | 11 |
| 16-42 | ENO1 | gi|46048768 | - | - | 9.1e-18 | 2.97421 | - | - | 232 | 8 |
| 16-43 | TXNDC4 | gi|50748830 | - | - | 0.0085 | 1.84483 | - | - | 92 | 3 |
| 16-49 | PPP1CB | gi|345323276 | - | - | 1.8e-22 | 1.75088 | 1.8e-22 | -1.61021 | 279 | 14 |
| 16-50 | HSPBP1 | gi|326935903 | - | - | 3.6e-05 | 1.61513 | 3.6e-05 | -1.98975 | 106 | 2 |
| 16-61 | DYNC1I2 | gi|55726736 | - | - | 3.6e-14 | 2.19624 | - | - | 196 | 6 |
| 16-62 | GARS | gi|71895709 | - | - | 1.4e-11 | 3.26323 | - | - | 170 | 13 |
| 16-65 | PSPC1 | gi|71895115 | - | - | 2.3e-18 | 2.27843 | 2.3e-1 | -2.04663 | 238 | 5 |
| 16-69 | PITPNB | gi|86129444 | - | - | 2.3e-17 | 100 | 2.3e-17 | -2.84167 | 228 | 6 |
| 21-01 | PDCL3 | gi|71895205 | - | - | - | - | 2.3e-05 | -2.09499 | 108 | 6 |
| 22-02 | IMMT | gi|57530041 | - | - | - | - | 1.1e-57 | -3.00226 | 631 | 19 |
| 22-12 | CCT7 | gi|71895883 | - | - | - | - | 0.0059 | -2.08184 | 84 | 6 |
| 22-14 | ALDH7A1 | gi|118104602 | - | - | - | - | 0.00022 | -1.58333 | 98 | 9 |
| 22-15 | PRKAR1A | gi|56119042 | - | - | - | - | 7.2e-21 | -2.51897 | 263 | 5 |
| 22-21 | SEPT2 | gi|57525156 | - | - | - | - | 5.8e-30 | -1.60527 | 354 | 8 |
| 22-23 | STRAP | gi|57525428 | - | - | - | - | 7.2e-10 | -3.11417 | 153 | 3 |
| 22-24 | HNRNPC | gi|119586801 | - | - | - | - | 0.0044 | -3.00556 | 85 | 2 |
| 22-37 | ALDH6A1 | gi|50748470 | - | - | - | - | 3.6e-13 | -3.11175 | 186 | 8 |
| 22-39 | RUVBL1 | gi|57524964 | - | - | - | - | 5.8e-18 | -2.28847 | 234 | 5 |
| 22-46 | PPP2CA | gi|45384108 | - | - | - | - | 3.6e-15 | -3.49885 | 206 | 10 |

**^a^** to **^g^** refer to the corresponding footnotes in Table 1.

**Supplementary Table S3. Pairwise comparison of DE proteins in CEF at 36 hpi with the H5N1 virus rNA-wt or rSD20.**

| **Spot**  **ID^a^** | **Protein name (Abbreviation)** | **Accession**  **No^b^** | **Differentially expressed proteins identified in CEF between groups** | | | | | | **Protein**  **score^d^** | **Matched peptide^e^** |
| --- | --- | --- | --- | --- | --- | --- | --- | --- | --- | --- |
|  |  |  | **rNA-wt and Mock** | | **rSD20 and Mock** | | **rNA-wt and rSD20** | |  |  |
|  |  |  | ***P-*value** | **Ratio^c^** | ***P-*value** | **Ratio** | ***P-*value** | **Ratio** |  |  |
| 27-07 | Vimentin | gi|114326309 | 7.2e-25 | -2.82649 | -^f^ | - | - | - | 303 | 8 |
| 27-11 | PSPC1 | gi|71895115 | 2.3e-18 | -1.83600 | - | - | - | - | 238 | 5 |
| 27-13 | PKM2 | gi|45382651 | 1.3 | -2.30525 | 1.3 | -100^g^ | - | - | 87 | 9 |
| 27-19 | CKAP4 | gi|118082813 | 5.8e-15 | -100 | 5.8e-15 | -1.71872 | 5.8e-15 | -1.84109 | 204 | 8 |
| 27-20 | RUVBL2 | gi|148230609 | 7.2e-05 | -2.17571 | - | - | - | - | 103 | 10 |
| 27-22 | CALU | gi|47498076 | 3.6e-06 | -3.26964 | 3.6e-06 | -1.53459 | - | - | 116 | 5 |
| 27-25 | ATXN3 | gi|45383440 | 1.8e-09 | -1.64882 | - | - | - | - | 149 | 7 |
| 27-26 | TARDBP | gi|71894865 | 1.4e-10 | -1.78863 | - | - | - | - | 160 | 3 |
| 27-27 | RPSA | gi|308081909 | 9.1e-36 | -20.9744 | 9.1e-36 | -14.2236 | - | - | 412 | 8 |
| 27-28 | SPARC | gi|45383337 | 5.8e-16 | -6.99618 | 5.8e-16 | -6.29835 | - | - | 214 | 7 |
| 27-29 | CTSD | gi|45384002 | 1.8e-05 | -4.41233 | 1.8e-05 | -2.03543 | - | - | 109 | 2 |
| 27-30 | HNRPA3 | gi|118093536 | 0.00017 | -2.99161 | - | - | - | - | 99 | 3 |
| 27-31 | **SAE1** | gi|118125845 | 0.00015 | -2.82337 | 0.00015 | -2.23239 | - | - | 100 | 2 |
| 27-32 | CAPG | gi|118105438 | 5.8e-08 | -1.72002 | - | - | - | - | 134 | 2 |
| 27-33 | SEPT2 | gi|57525156 | 5.8e-30 | -2.73851 | - | - | - | - | 354 | 8 |
| 27-34 | CNN3 | gi|50751284 | 5.8e-07 | -1.62784 | 5.8e-07 | -1.72513 | - | - | 124 | 11 |
| 27-35 | STRAP | gi|57525428 | 7.2e-10 | -2.76061 | 7.2e-10 | -2.44387 | - | - | 153 | 3 |
| 27-36 | HNRNPC | gi|119586801 | 0.0044 | -2.39232 | 0.0044 | -1.60089 | - | - | 85 | 2 |
| 27-37 | HNRNPC | gi|119586801 | 0.00017 | -100 | 0.00017 | -3.95078 | - | - | 99 | 3 |
| 27-38 | **CRISPLD1** | [gi|45382819](http://www.matrixscience.com/cgi/protein_view.pl?file=../data/20110917/FtocmesOL.dat&hit=8) | 62 | -2.62489 | - | - | - | - | 44 | 5 |
| 27-39 | LASP1 | gi|293601661 | 1.4e-11 | -2.08944 | - | - | - | - | 170 | 6 |
| 27-43 | WDR61 | gi|94536819 | 0.00023 | -2.24299 | - | - | - | - | 98 | 5 |
| 27-44 | HSP90A | gi|157954047 | 7.2e-20 | -2.37816 | 7.2e-20 | -1.58329 | - | - | 253 | 8 |
| 27-45 | PRKCSH | gi|345326432 | 0.016 | -5.36157 | 0.016 | -2.21476 | - | - | 79 | 3 |
| 27-47 | ACTG1 | gi|56119084 | 4.5e-17 | -100 | 4.5e-1 | -100 | - | - | 225 | 7 |
| 27-49 | Vimentin | gi|114326309 | 1.4e-29 | -1.88234 | 1.4e-29 | -1.58283 | - | - | 350 | 11 |
| 27-50 | FKBP9 | gi|45382327 | 5.8e-27 | -2.32492 | 5.8e-27 | -1.67835 | - | - | 324 | 9 |
| 27-51 | Vimentin | gi|114326309 | 1.4e-18 | -1.65879 | - | - | - | - | 240 | 11 |
| 27-52 | PRKAR1A | gi|56119042 | 7.2e-21 | -2.00653 | 7.2e-21 | -1.51996 | - | - | 263 | 5 |
| 27-53 | TRA2A | gi|57530708 | 5.7e-10 | -2.1912 | - | - | - | - | 154 | 4 |
| 27-55 | NACA | gi|209736954 | 4.6e-10 | -2.24779 | - | - | - | - | 155 | 4 |
| 27-57 | EF1D | gi|118087445 | 2.9e-1 | -100 | - | - | - | - | 197 | 6 |
| 28-05 | HSPA5 | gi|45382769 | 4.6e-27 | 3.63355 | - | - | - | - | 325 | 12 |
| 28-07 | Moesin | gi|50513540 | 0.0013 | 1.66711 | - | - | - | - | 90 | 8 |
| 28-08 | FAM114A2 | gi|50755009 | 1.1e-21 | 1.74559 | - | - | - | - | 271 | 5 |
| 28-09 | nucleocapsid ( NP) | gi|94483633 | 0.022 | 100 | - | - | - | - | 76 | 4 |
| 28-15 | TXNDC4 | gi|50736203 | 3.6e-05 | 2.98086 | - | - | - | - | 106 | 4 |
| 28-17 | Vimentin | gi|114326309 | 5.8e-32 | 3.97762 | 5.8e-32 | 3.3451 | - | - | 374 | 15 |
| 28-20 | NPM1 | gi|212456 | 1.1e-08 | 1.74704 | - | - | - | - | 141 | 2 |
| 28-23 | PPP1CC | gi|57525187 | 2.9e-19 | 6.54404 | 2.9e-19 | 5.47431 | - | - | 247 | 12 |
| 28-24 | CAPZA1 | gi|297787503 | 1.8e-17 | 1.97032 | 1.8e-17 | 1.88101 | - | - | 229 | 8 |
| 28-25 | Regucalcin (RGN) | gi|45382019 | 9.1e-08 | 100 | - | - | - | - | 132 | 7 |
| 28-26 | SRSF1 | gi|82233969 | 1.4e-33 | 3.31431 | - | - | - | - | 390 | 11 |
| 28-28 | PDHB | gi|310750374 | 1.1e-16 | 3.02946 | 1.1e-16 | 2.08361 | - | - | 221 | 5 |
| 28-27 | PDCL3 | gi|71895205 | 2.3e-05 | 2.05583 | - | - | - | - | 108 | 6 |
| 28-29 | PSMA3 | gi|57529899 | 3.6e-21 | 2.9959 | 3.6e-21 | 2.35677 | - | - | 266 | 11 |
| 28-30 | Vimentin | gi|57240090 | 3.6e-37 | 3.63858 | 3.6e-37 | 3.74892 | - | - | 426 | 15 |
| 28-31 | Vimentin | gi|57240090 | 7.2e-20 | 4.68057 | 7.2e-20 | 3.80343 | - | - | 253 | 10 |
| 28-32 | PRDX4 | gi|118084001 | 4.5e-22 | 100 | 4.5e-22 | 100 | - | - | 275 | 9 |
| 28-34 | YWHAB | gi|57529350 | 9.1e-08 | 2.49387 | 9.1e-08 | 2.13389 | - | - | 132 | 7 |
| 28-36 | LMNB2 | gi|45384202 | 9.1e-16 | 3.69111 | 9.1e-16 | 3.14255 | - | - | 212 | 11 |
| 28-37 | PRDX4 | gi|118084001 | 1.1e-29 | 100 | 1.1e-29 | 100 | - | - | 351 | 12 |
| 28-38 | Influenza NS1 | gi|50296338 | 3e-14 | 3.91946 | 3e-14 | 4.03709 | - | - | 207 | 5 |
| 28-39 | CALB1 | gi|45382893 | 9.1e-09 | 2.61528 | - | - | 9.1e-09 | 1.69141 | 142 | 5 |
| 28-40 | CBX1 | gi|45383494 | 0.00045 | 3.71134 | - | - | - | - | 95 | 2 |
| 28-42 | ITPA | gi|50751047 | 2.3e-11 | 100 | - | - | - | - | 168 | 7 |
| 28-50 | ACTL6A | gi|118095278 | 2.3e-16 | 5.82293 | - | - | - | - | 218 | 7 |
| 28-43 | CHP | gi|56118996 | 3.6e-21 | 100 | - | - | - | - | 266 | 5 |
| 28-44 | Vimentin | gi|57240089 | 3.6e-19 | 3.82605 | - | - | - | - | 246 | 8 |
| 28-45 | Vimentin | gi|114326309 | 5.8e-13 | 3.15823 | - | - | - | - | 148 | 9 |
| 28-49 | CSTF2 | gi|224097289 | 0.0017 | 5.21311 | 0.0017 | 6.36314 | - | - | 89 | 5 |
| 28-52 | Influenza NS1 | gi|262400876 | 4.8e-14 | 2.00653 | 4.8e-14 | 50.8553 | - | - | 205 | 6 |
| 29-12 | PPP2R1A | gi|327280792 | - | - | 1.8e-35 | -1.66518 | - | - | 168 | 7 |
| 30-14 | LMNA | gi|45384214 | - | - | 2.9e-25 | 2.30665 | 2.9e-25 | -1.72487 | 307 | 22 |
| 30-16 | HSPA2 | gi|55742654 | - | - | 0.00024 | 100 | 0.00024 | -100 | 98 | 5 |
| 30-17 | SDHA | gi|3851616 | - | - | 9.1e-07 | 1.75655 | - | - | 122 | 6 |
| 30-18 | HSPA8 | gi|190576828 | - | - | 0.04 | 5.94383 | 0.04 | -4.04124 | 76 | 5 |
| 30-19 | HSP75 | gi|57525126 | - | - | 4.6e-17 | 1.69483 | - | - | 225 | 10 |
| 30-23 | PSPC1 | gi|71895115 | - | - | 2.9e-19 | 1.86238 | 2.9e-19 | -2.13783 | 247 | 11 |
| 30-25 | FKBP4 | gi|57525441 | - | - | 7.2e-06 | 100 | 7.2e-06 | -100 | 113 | 6 |
| 30-31 | ACTG1 | gi|56119084 | - | - | 9.1e-18 | 2.89076 | - | - | 232 | 4 |
| 30-37 | PITPNB | gi|86129444 | - | - | 2.3e-17 | 100 | - | - | 228 | 6 |
| 30-46 | TPM3 | gi|53129586 | - | - | 1.4e-11 | 2.76210 | 1.4e-11 | -3.33766 | 170 | 8 |
| 30-48 | Nucleoprotein (NP) | gi|440246 | - | - | 4.8e-23 | 2.44459 | 4.8e-23 | -1.51591 | 295 | 9 |
| 30-49 | ALDH6A1 | gi|50748470 | - | - | 3.6e-13 | 3.22303 | 3.6e-13 | -2.01413 | 186 | 8 |
| 30-50 | Nucleoprotein (NP) | gi|94483633 | - | - | 7.6e-14 | 1.88659 | - | - | 203 | 8 |
| 30-52 | TXNDC5 | gi|57530789 | - | - | 1.1e-22 | 2.46603 | - | - | 281 | 6 |
| 31-02 | DCTN2 | gi|45382201 |  | - | - | - | 1.4e-14 | -17.7082 | 200 | 6 |
| 32-07 | HSP90B1 | gi|63509 | - | - | - | - | 3.6e-12 | -3.14319 | 176 | 14 |
| 32-32 | LMNA | gi|45384214 | - | - | - | - | 9.1e-14 | -100 | 192 | 16 |

**^a^** to **^g^** refer to the corresponding footnotes in Table 1.

**Supplementary Table S4.** Sequences of primer pairs used for qRT-PCR.

| Genes | Forward primer(5’-3’) | Reverse primer(5’-3’) |
| --- | --- | --- |
| HSP70 | GGCACCATCACTGGGCTTA | TCCAAGCCATAGGCAATAGCA |
| GAPDH | CACTGTCAAGGCTGAGAACGG | GGAGCTGAGATGATAACACGCTT |
| Vimentin | ACAGCACATCCAAATCGATATG | TTCGGAGAGATCTGCAAATTTG |
| LMNB2 | TTCCCGAGGCAAGAGACGAC | GCCCAGCCCTGAGGACATACT |
| ENO1 | AGCGGAGCGGTGTTCAAGAT | CAGCCAGAGATACGCCCAAGAT |
| HSP60 | ACAGCCAAAGGGCAGAAATG | TTCAAGACTAGAGTGCTGAGGGC |
| TGF-β | CCGACACGCAGTACACCAAG | CAGGCACGGACCACCATATT |
| Smad2 | GCCGAGTGCCTCAGTGATAGC | TGAGCCAGAAGAGCAGCAAACT |

**
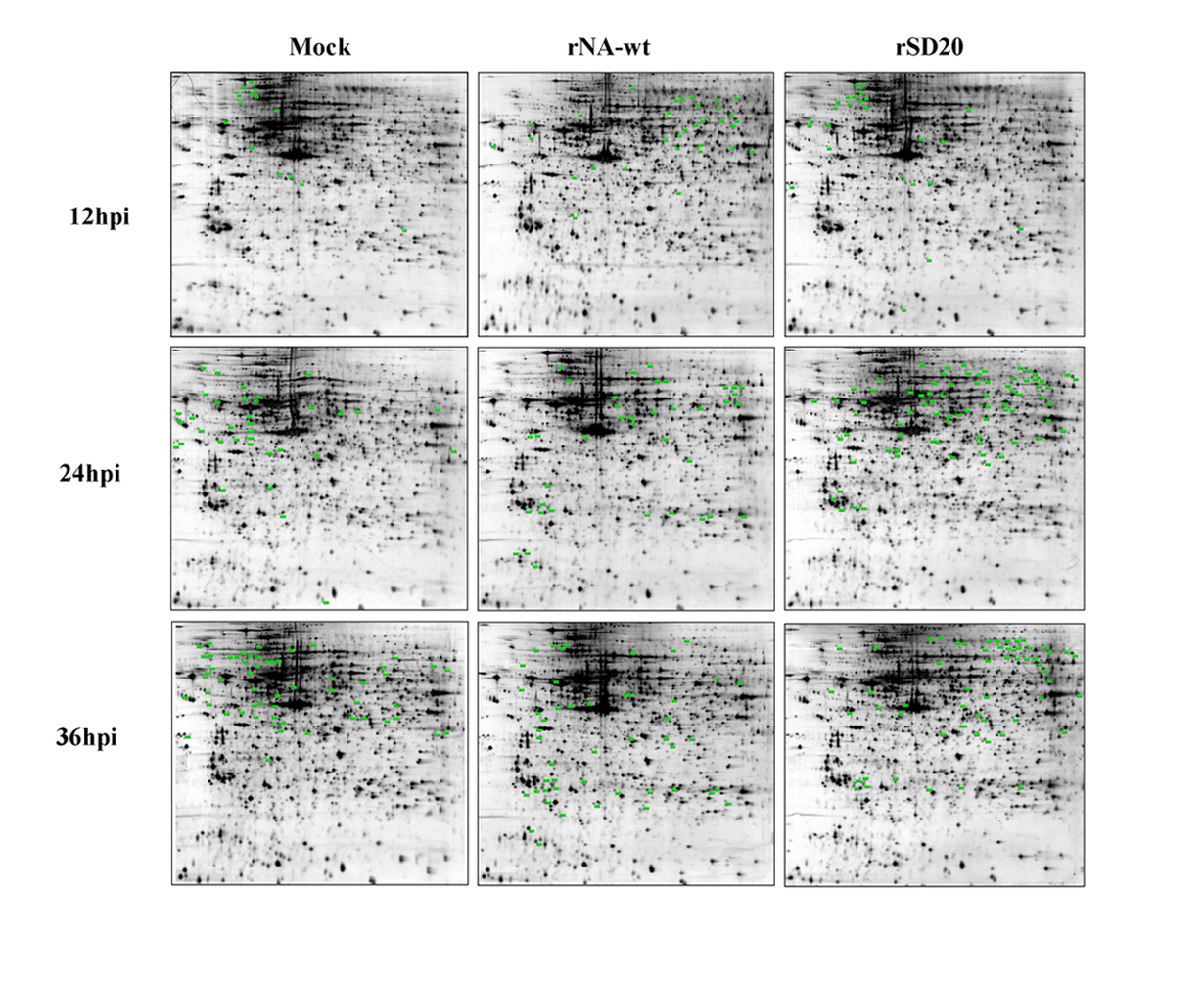
**

**Supplementary Fig S1.** Proteome maps of DE proteins in CEF cells after infection with H5N1 influenza viruses. Groups of CEF cells were infected with the indicated viruses or with PBS and were collected at 12, 24 and 36 hpi. The DE proteins detected are marked by arrows in green and their numbers are consistent with those shown in Tables 1, 2 and 3. The inoculations are indicated at the top and the time points for CEF cells collection are shown on the left. Images were shown as the representative generated from samples in each group.

**
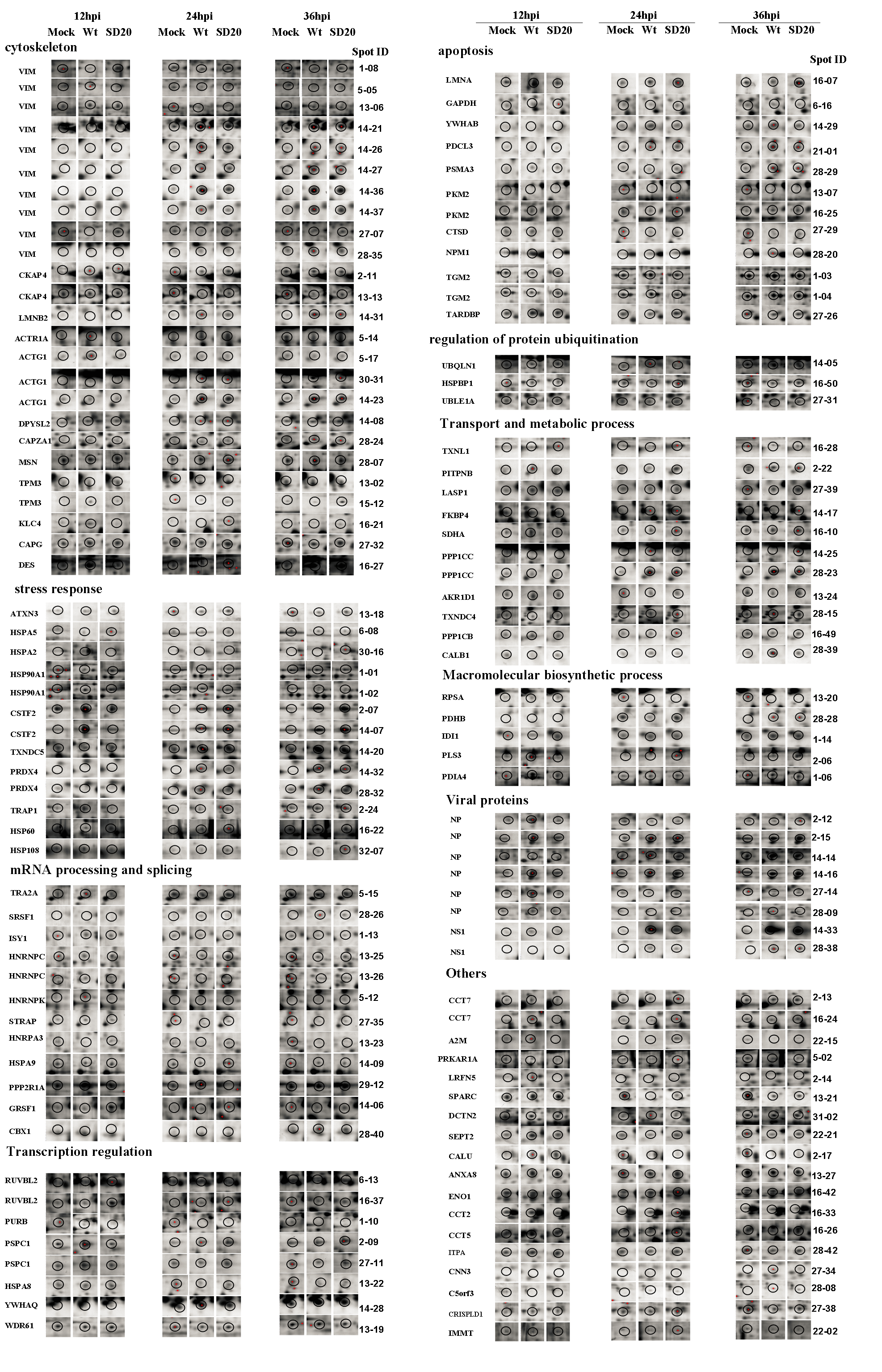
**

**Supplementary Fig S2.** Dynamic 2-DE profiles of the DE proteins in the H5N1-infected CEF cells. Circles indicate the DE protein spots. CK indicates the regulated protein in uninfected cells. rNA-wt and rSD20 indicated the protein spots of rNA-wt and rSD20 infected cells respectively.


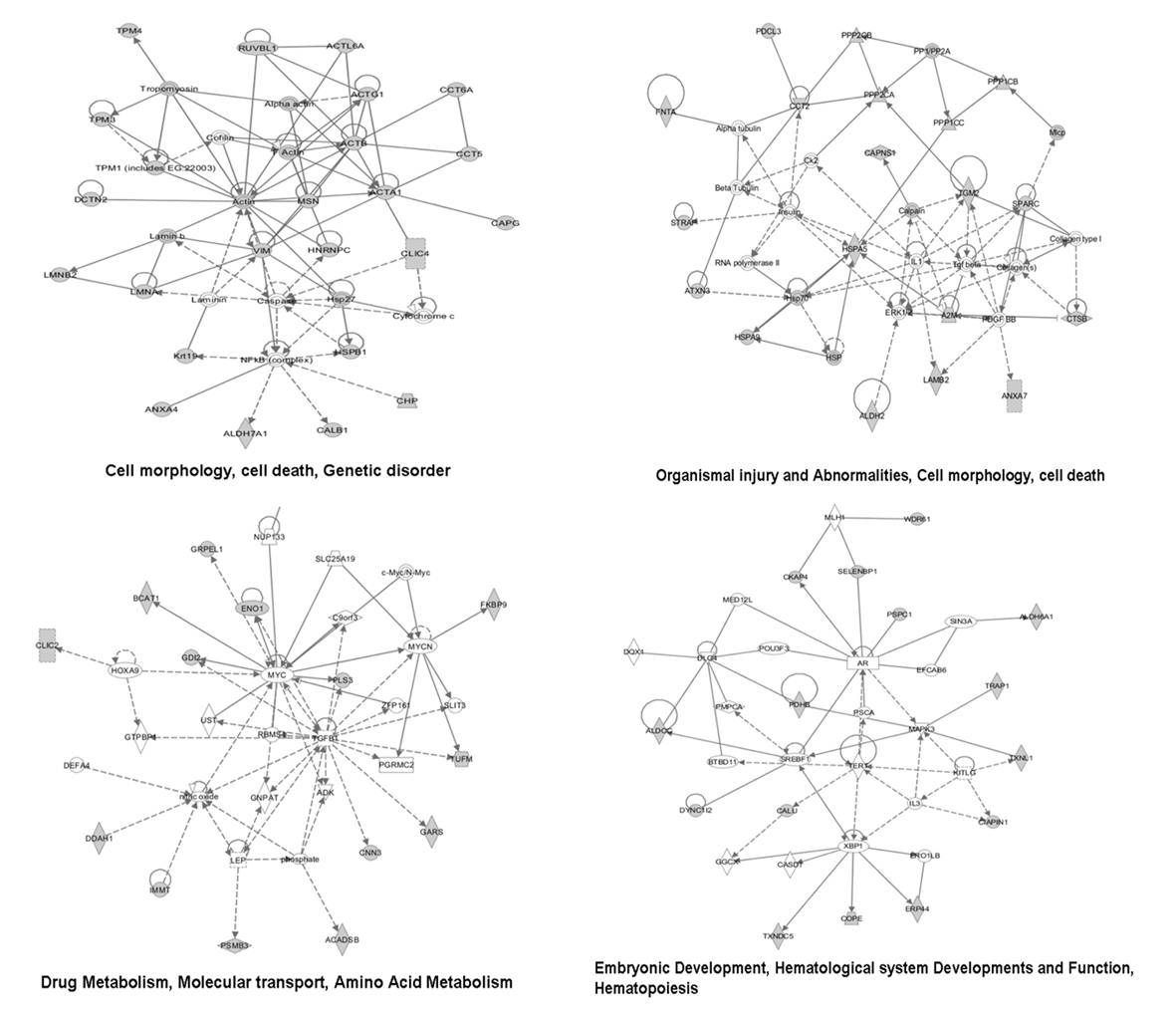


**Supplementary Fig S3**. The top pathways of total DE proteins by IPA.

**Supplementary Fig S4.** Western blot analysis of β-actin


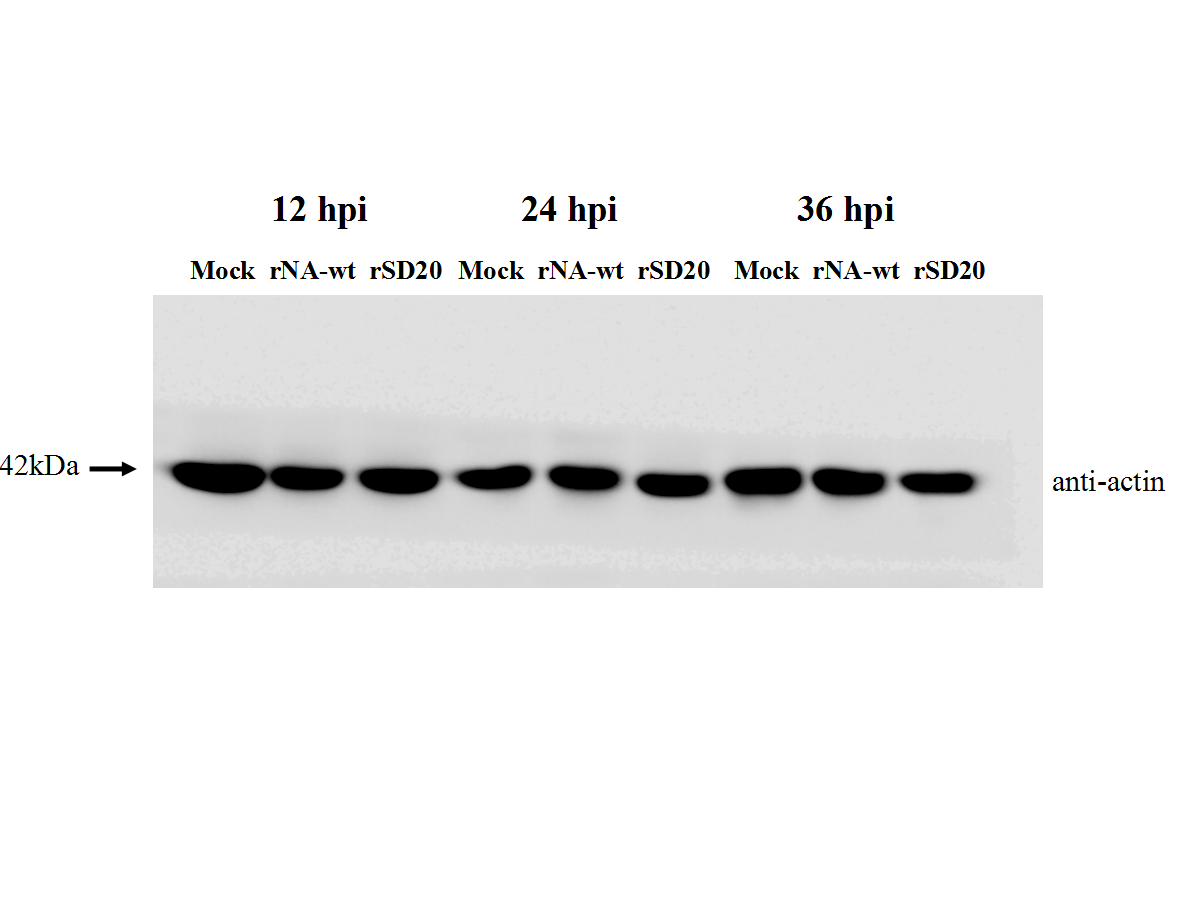


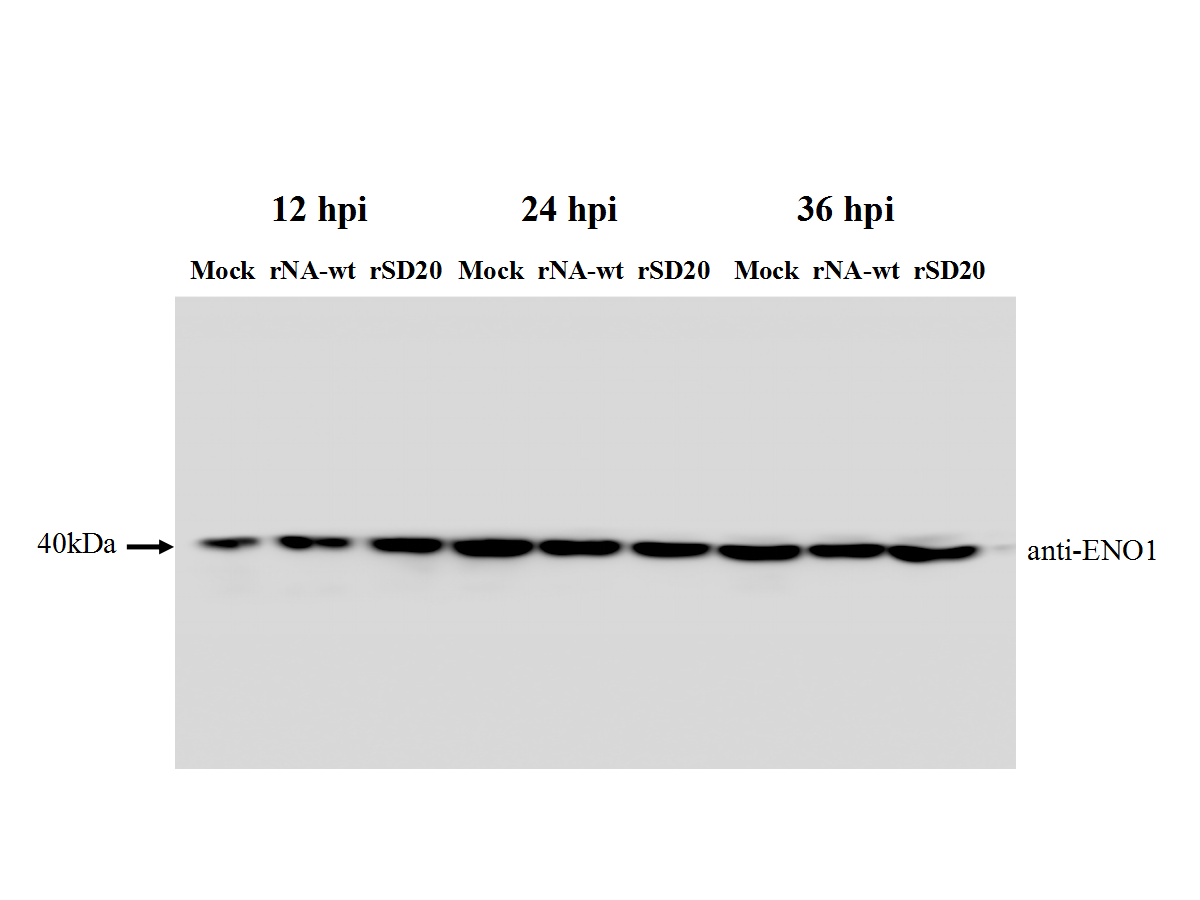
**Supplementary Fig S5.** Western blot analysis of ENO1

**Supplementary Fig S6.** Western blot analysis of GAPDH


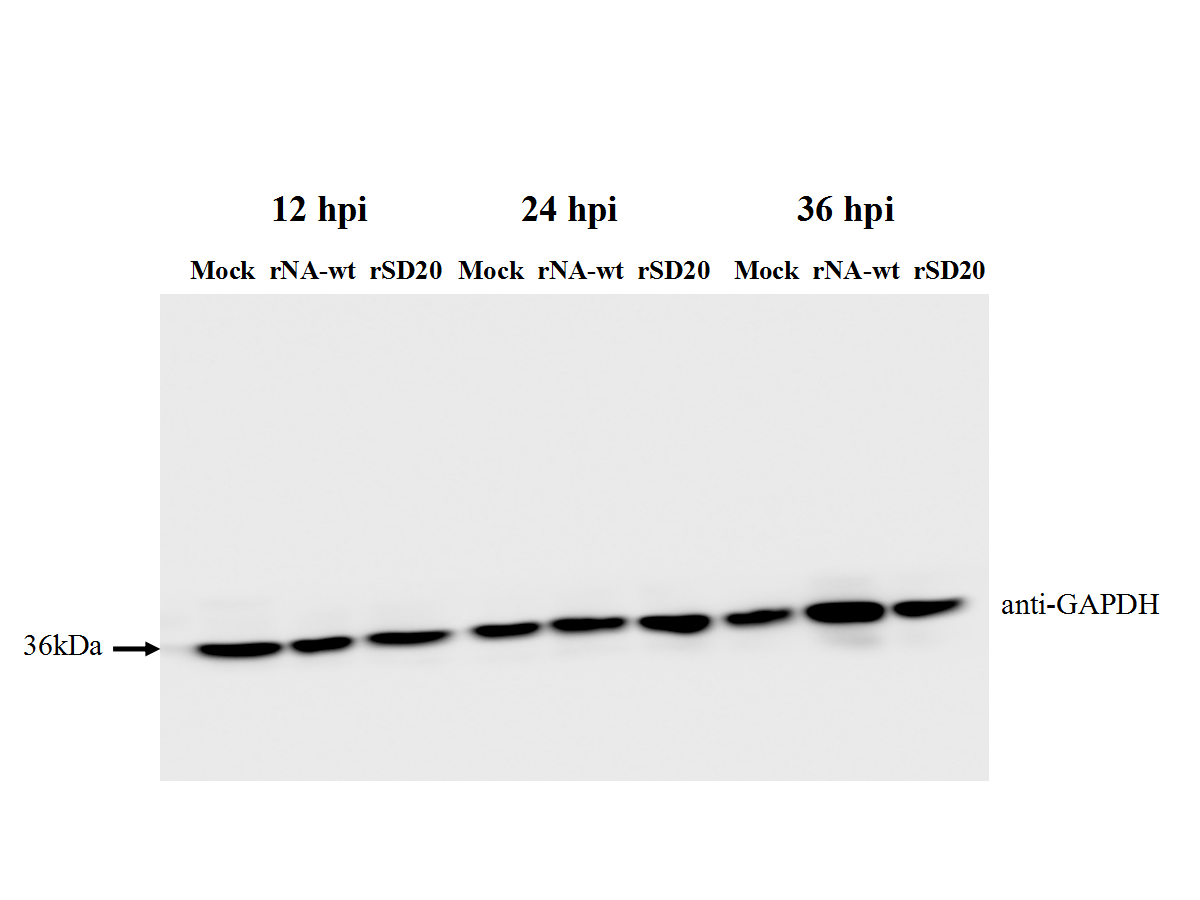


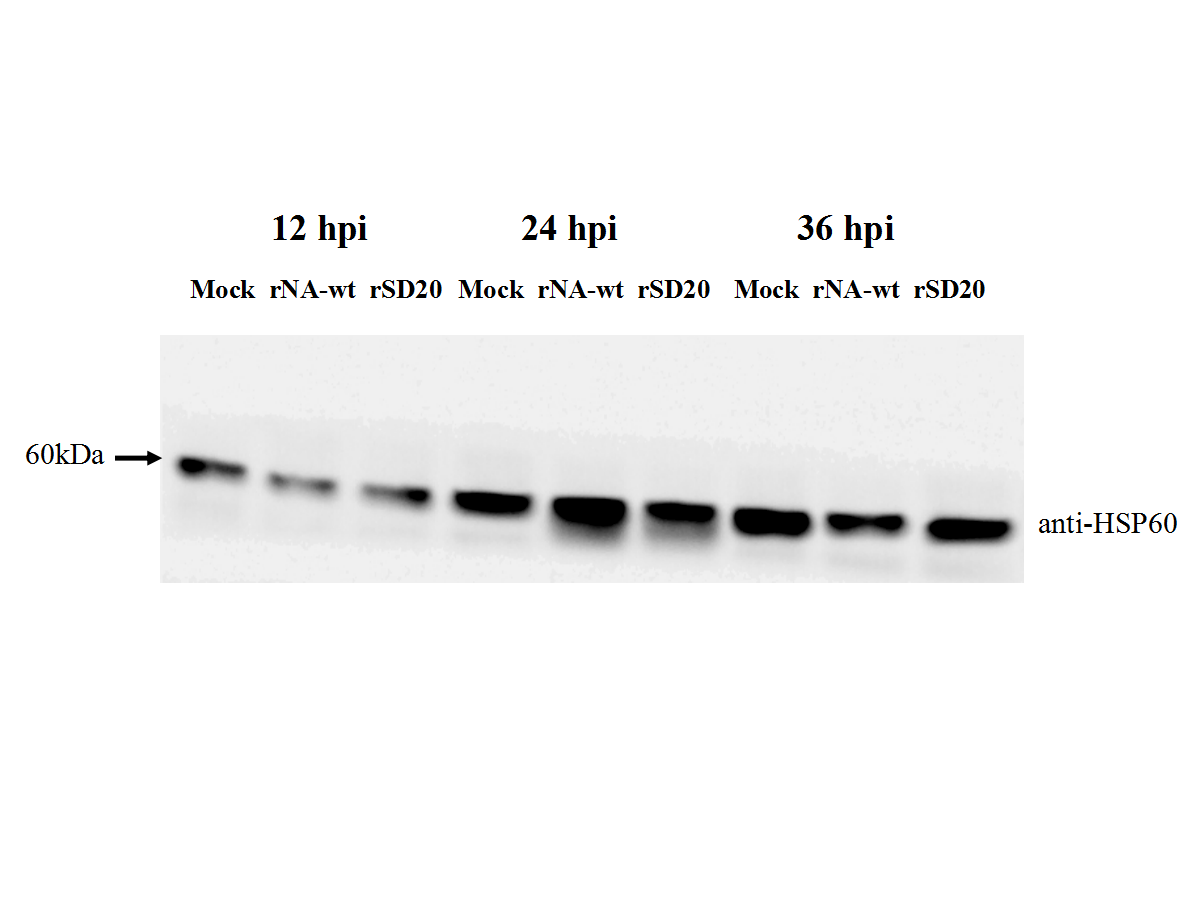
**Supplementary Fig S7.** Western blot analysis of HSP60

**Supplementary Fig S8.** Western blot analysis of HSP70


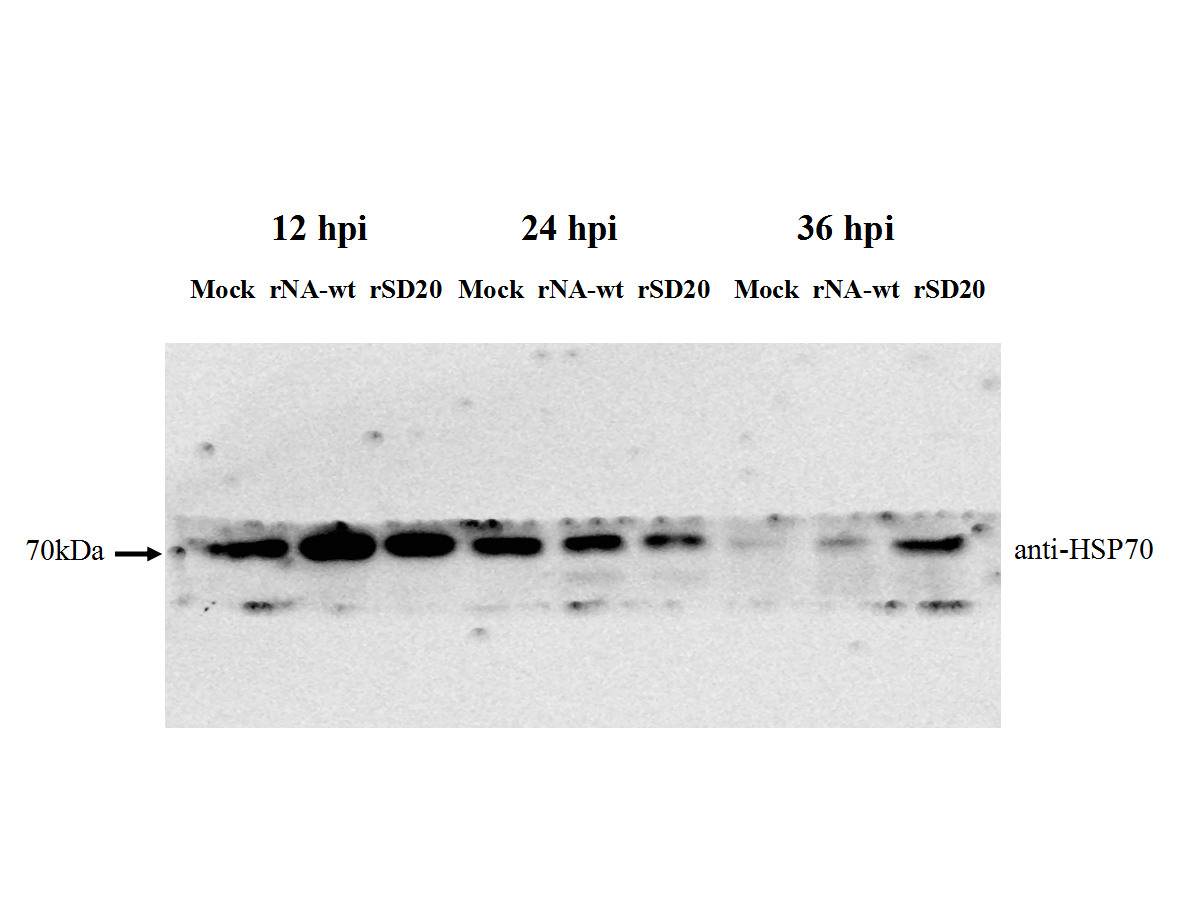


**Supplementary Fig S9.** Western blot analysis of PSMA3


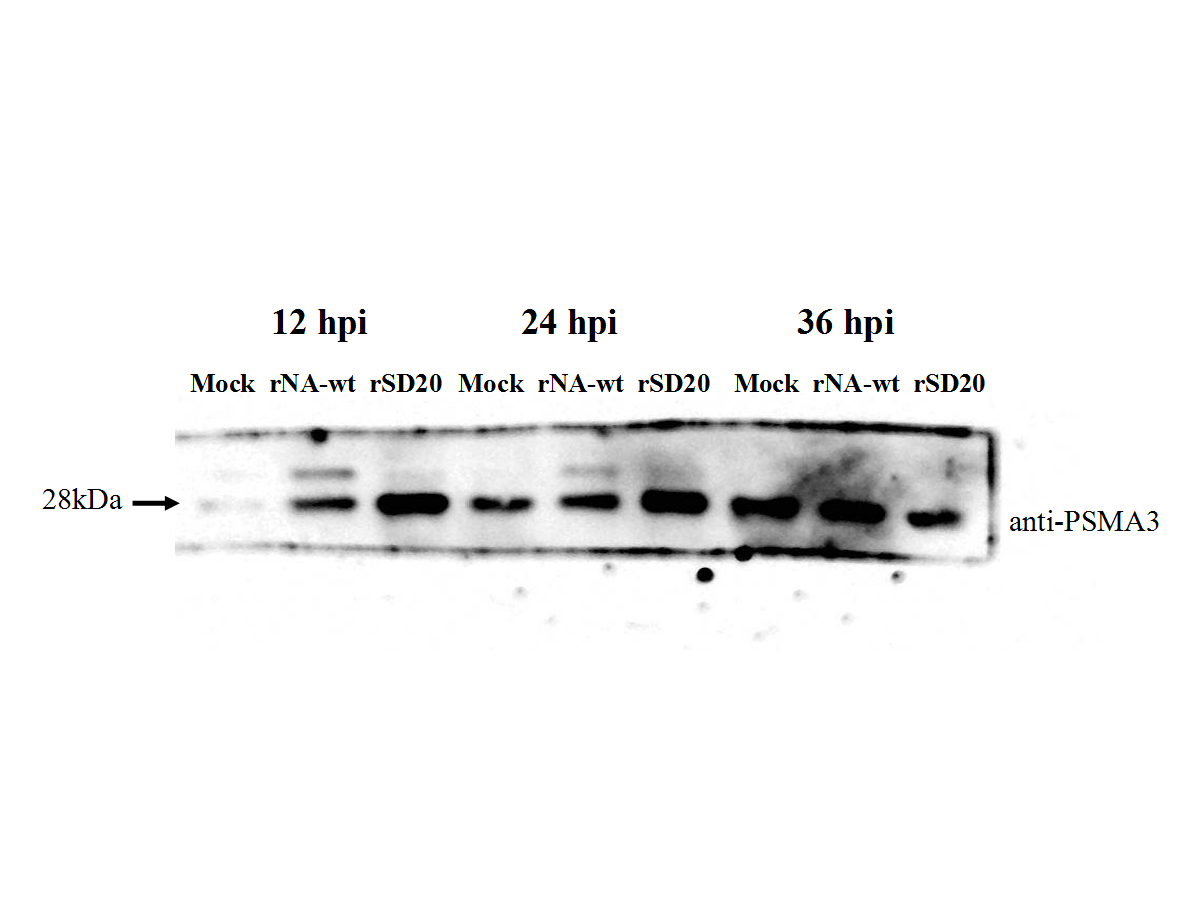


**Supplementary Fig S10.** Western blot analysis of VIM


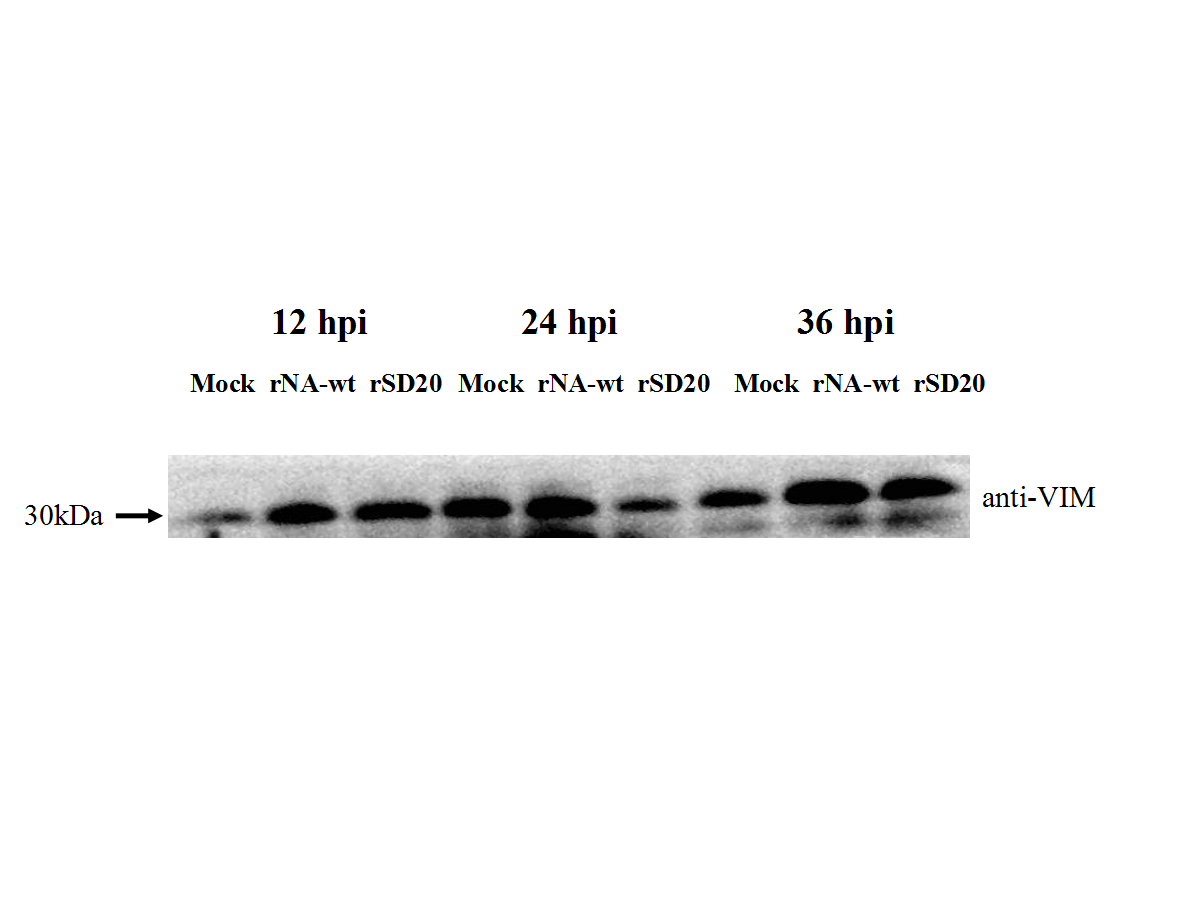

Supplement: Supplementary Information [file srep40698-s1.doc]
